# A Continuous-Time Dynamic Factor Model for Intensive Longitudinal Data Arising from Mobile Health Studies

**DOI:** 10.1017/psy.2025.10023

**Published:** 2025-06-16

**Authors:** Madeline R. Abbott, Walter H. Dempsey, Inbal Nahum-Shani, Cho Y. Lam, David W. Wetter, Jeremy M. G. Taylor

**Affiliations:** 1 Department of Biostatistics, University of Michigan, Ann Arbor, MI, USA; 2 Institute for Social Research, University of Michigan, Ann Arbor, MI, USA; 3 Department of Population Health Sciences and Huntsman Cancer Institute, University of Utah, Salt Lake City, UT, USA

**Keywords:** dynamic factor model, intensive longitudinal data, mobile health, Ornstein-Uhlenbeck stochastic process

## Abstract

Intensive longitudinal data (ILD) collected in mobile health (mHealth) studies contain rich information on the dynamics of multiple outcomes measured frequently over time. Motivated by an mHealth study in which participants self-report the intensity of many emotions multiple times per day, we describe a dynamic factor model that summarizes ILD as a low-dimensional, interpretable latent process. This model consists of (i) a measurement submodel—a factor model—that summarizes the multivariate longitudinal outcome as lower-dimensional latent variables and (ii) a structural submodel—an Ornstein–Uhlenbeck (OU) stochastic process—that captures the dynamics of the multivariate latent process in continuous time. We derive a closed-form likelihood for the marginal distribution of the outcome and the computationally-simpler sparse precision matrix for the OU process. We propose a block coordinate descent algorithm for estimation and use simulation studies to show that it has good statistical properties with ILD. Then, we use our method to analyze data from the mHealth study. We summarize the dynamics of 18 emotions using models with one, two, and three time-varying latent factors, which correspond to different behavioral science theories of emotions. We demonstrate how results can be interpreted to help improve behavioral science theories of momentary emotions, latent psychological states, and their dynamics.

## 1 Introduction

Intensive longitudinal data (ILD) can capture rapid changes in outcomes over time. Often in mobile health (mHealth) studies, many longitudinal outcomes are measured with the aim of understanding the temporal dynamics of unobservable constructs related to mental or physical health. Our work is motivated by an observational mHealth study in which the intensity of emotions was collected over time. Participants ( 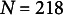 ) self-reported the intensity of 18 different emotions up to four times per day over 10 days, resulting in a substantial quantity of rich data. For behavioral scientists, understanding the temporal dynamics of the latent psychological states that underlie these emotions—and how well these emotions measure the specific latent states—is of scientific interest.

The volume and complexity of ILD, however, make them challenging to analyze since longitudinal outcomes are often measured irregularly across many individuals. Thus statistical methods must be able to handle the irregular spacing of this high volume of data. At the same time, the frequent measurements in ILD create opportunities to discover new information, particularly if the latent constructs of interest vary rapidly. We present a dynamic factor model that is motivated by the need to model multiple longitudinal outcomes measured frequently over time in a flexible yet interpretable manner. The dynamic factor model, which is similar to that described in Tran et al. (2021a), consists of two submodels: (i) a measurement submodel—a factor model—that summarizes the multiple observed longitudinal outcomes as lower-dimensional latent factors and (ii) a structural submodel—an Ornstein–Uhlenbeck (OU) stochastic process—that captures the evolution of the multiple correlated latent factors over time. Together, these components of our dynamic factor model are flexible enough to capture the volatility in the longitudinal outcomes while avoiding use of a non-parametric or other many-parameter model that may inhibit interpretability. The low-dimensional nature of the structural submodel also greatly reduces computational complexity, as opposed to fitting a high-dimensional stochastic process directly to the observed outcomes. The dynamics of the latent factors are an important aspect of the model, in contrast to the measurement focus of the classic P-technique factor analysis approach, which assumes time-invariant latent factors (Molenaar & Nesselroade, 2009).

One standard approach to modeling changes in multiple correlated longitudinal variables is to use an autoregressive (AR) model, or a vector AR (VAR) model if data are multivariate. AR and VAR processes appear frequently in the behavioral science literature, where they are used to model phenomena such as depressive symptoms (Groen et al., 2019; Snippe et al., 2017) and emotions (Krone et al., 2018). These types of processes have been used widely to model both observed outcomes as well as latent variables. For example, Dunson (2003); Cui & Dunson (2014) and Tran et al. (2021b) have proposed related methods in which observed longitudinal outcomes are summarized as time-varying lower-dimensional latent variables. The correlation of these latent variables is then modeled with AR or VAR processes. AR and VAR models, however, are specified for data with equally spaced measurement occasions. This situation is often not realistic in the case of ILD, which generally consists of irregularly-measured outcomes, and can lead to biased estimates in cases where the assumption is made but does not hold.

**Figure 1:**
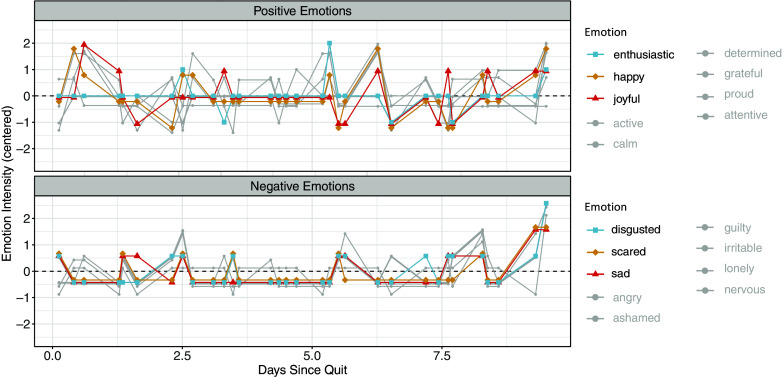
Responses to the EMA questions over time for one participant in the mHealth study, separated by positive and negative emotions. In this plot, a subset of three positive emotions and three negative emotions are highlighted solely for illustrative purposes; all 18 emotions are later included in the model. Note both the high correlation and volatility of these longitudinal outcomes over time.

Mixed models have been proposed as alternatives to discrete-time processes for modeling the evolution of latent variables over time, and have been previously used in combination with factor models. Unlike the AR and VAR processes, mixed models do not require equally spaced measurement occasions. Existing work has focused both on the development of mixed models for modeling the evolution of a single latent factor over time (e.g., Proust et al., 2006; Proust-Lima et al., 2013; Roy & Lin, 2000) or multiple latent factors (e.g., Liu et al., 2019; Wang et al., 2013). Overall, mixed model-based approaches—including both traditional mixed models and their extensions to multi-level factor models—are useful tools for capturing smooth trends in latent factors and understanding within- and between-individual variation. However, they may have trouble capturing changes that happen rapidly (e.g., volatile changes in psychological states, as seen in the positive emotions around day 6 in Figure 1, or in other ILD).

The OU process, which is a continuous-time analog of the commonly-used AR or VAR process, is a stochastic process well-suited for capturing volatility over time. Existing work has frequently focused on using the OU process or integrated OU process to model longitudinal outcomes that have been directly observed (or observed with measurement error); e.g., Oravecz et al., 2009, 2016; Sy et al., 1997; Taylor et al., 1994.

Most closely related to our proposed approach is the work in Tran et al. (2021a). Like us, the authors propose a longitudinal latent variable model that consists of two parts: a measurement submodel to summarize observed outcomes as lower dimensional latent factors and an OU process as the structural submodel for the latent factors. While we differ in the exact specification of the measurement submodel, our chosen models are related. The key distinction between this existing work and the work presented in this manuscript lies in the approach to estimation and inference. Tran et al. (2021a) take a Bayesian approach, which uses a form of the likelihood that requires sampling values of the latent factors at each measurement occasion. In the ILD setting, we need approaches that can scale to large numbers of repeated measurements. Here, we choose to work in the frequentist framework and directly maximize the marginal log-likelihood of the observed longitudinal outcome by integrating out latent variables, resulting in a method more suitable for ILD. While Tran et al. (2021a) present an approach that relies on algebraic constraints to fit models with two or three latent factors, our maximum-likelihood approach enables us to easily extend our model to larger numbers of latent factors through the use of penalties, rather than algebraic constraints. Finally, although we—like Tran et al. (2021a)—assume that the number of latent factors is known, our work additionally investigates the use of information criteria to select the true model among models with misspecified numbers of latent factors in a simulation study. The marginal log-likelihood of the observed data that we use here better facilitates the use of information criteria to compare models with different numbers of latent factors, as opposed to a version of the likelihood that conditions on the latent variables (see Merkle et al., 2019 for more discussion of marginal vs. conditional likelihoods for factor models).

In this work, we build on the model from Tran et al. (2021a) by developing and evaluating the performance of an efficient estimation algorithm that has the computational ability to handle ILD. Our work enables the analysis of high-dimensional ILD using low-dimensional stochastic latent variable models; as a result, these models can be used to understand how well observed longitudinal outcomes measure underlying states, how correlated these latent states are over time, and how much of the variation in the longitudinal outcome is related to short-term changes within an individual vs. longer-term differences across individuals. Designed specifically for the ILD setting, our novel methodological contributions include (i) a closed-form likelihood for the marginal distribution of the observed outcome, (ii) the derivation of the computationally-simpler sparse precision matrix for the multivariate OU process, (iii) identifiability constraints imposed via scaling constants, and (iv) a block coordinate descent algorithm for estimation and inference in a maximum likelihood framework.

The remainder of this article is organized as such: In Section 2, we describe the motivating ILD from an mHealth study; in Section 3, we present the model and our novel methodological contributions; in Section 4, we demonstrate the performance of our method via simulation; in Section 5, we use our method to analyze intensive longitudinal emotion data collected in an mHealth study; and in Section 6, we provide a discussion.

## 2 Motivating data

The ILD motivating this work consist of self-reported emotional states collected in an observational mHealth study (Potter et al., 2023). Over a period of 10 days, ecological momentary assessments (EMAs), which enable repeated sampling of individuals’ current states and contexts in real time, were used to frequently track participants’ emotions as they were experienced. Specifically, participants were prompted to respond to a series of questions sent to their smartphones multiple times per day at random occasions; the original study design intended for individuals to receive up to four EMAs per day. The EMAs contained a set of questions that assessed the current intensity of multiple emotions measured on a five-point Likert scale. We focus on a set of 18 emotions; these emotions are active, angry, ashamed, attentive, calm, determined, disgusted, enthusiastic, grateful, guilty, happy, irritable, joyful, lonely, nervous, proud, sad, and scared. These 18 emotions are a subset of the 23 emotions assessed at each EMA; we focus on 18 due to computational constraints. To arrive at this set of 18 emotions, we first fit a cross-sectional factor model and selected the subset of six emotions with the highest loadings to use in our dynamic factor model. Then, we gradually incorporated additional emotions from the remaining set of 17 until the computational cost of fitting the dynamic factor model became restrictive. The resulting data contain frequent measurements of a substantial number of longitudinal outcomes, where the number of measurement occasions per person ranges from 2 to 47 (mean = 17). The variability in total number of observations per person is due to a combination of intermittent non-response to the EMAs and dropout.

The high rate of measurement enables us to capture rapid changes in emotions—and thus different aspects of the latent psychological states—over time. Note that these data are the subset of the full study data that were available at the time of drafting this manuscript (*N* = 218 individuals). Additional details on the study can be found in Potter et al. (2023).

We illustrate the variability in these longitudinal outcomes in Figure 1, which shows the responses to emotion-related EMA questions over time for one participant in the study. The marginal distribution of each emotion is provided in the Supplementary Material (Supplementary Figure 3). Understanding the dynamics of individuals’ latent psychological states that underlie the measured responses, as well as investigating the appropriate number of latent states to summarize the observed responses, is of scientific interest among behavioral scientists.

## 3 Methods

In this section, we present the OU factor (OUF) model that jointly models multiple observed longitudinal outcomes (here, emotions) and the lower dimensional latent factors (representing, for example, psychological states) assumed to generate the observed longitudinal outcomes. The model consists of two submodels: a measurement submodel and a structural submodel.

### 3.1 Measurement submodel

Let 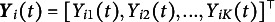 be a 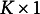 vector of measured longitudinal outcomes (e.g., emotions in the motivating data) for individual 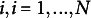 , at time *t*. Assume that individual *i* has longitudinal outcomes measured at 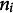 occasions (e.g., at 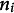 EMAs). Using the measurement submodel, we model the observed longitudinal outcome 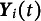 as (1)

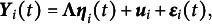

where 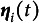 is a vector of *p* time-varying latent factors (where 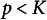 ); 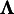 is a 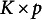 -dimensional time-invariant loadings matrix with elements 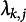 that captures the degree of association between the latent factors and observed longitudinal outcomes; 
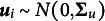
 is a vector of length *K* of random intercepts; and 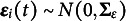 is a vector representing measurement error, where 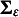 is assumed to be a diagonal matrix.

This model builds upon a standard factor model but also includes (i) a random intercept and (ii) a multivariate model for the evolution of the correlated latent processes 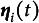 (described in the next section). This random intercept was previously introduced in Tran et al. (2021a). We assume that 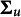 is diagonal, as we include this term to account for baseline differences across individuals, but then model the correlated change in outcomes through the structural submodel. Allowing a non-diagonal 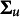 is possible, but we opt not to do so to avoid the substantial increase in computational cost associated with estimation of these extra parameters. If we were to allow the off-diagonal elements of 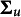 to vary, we would need to estimate 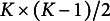 additional parameters; in our setting where 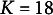 , this would be 153 additional parameters. In a different setting, however, domain knowledge may support the use of an alternative covariance matrix; information criteria could aid in selection among plausible covariance matrices. In the context of modeling emotions over time, we can interpret our random intercept as accounting for differences in psychological traits (i.e., a construct that is more stable within a person) while the dynamic latent factors capture changes in psychological state (i.e., a construct that varies more quickly; Schmitt & Blum, 2020). We similarly assume a diagonal structure for 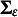 , implying that measurement error is independent for each emotion. While we make this decision for simplicity here, a different structure for 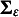 could be assumed in another context (although a more complicated structure would come at the expense of increased computation time).

We assume that the loadings are constant, which allows us to use the model to understand which of the longitudinal outcomes are most important to measure to capture the dynamics of the time-varying latent factors. Given that the motivating data span a period of only 10 days, assuming constant loadings is reasonable in our setting. In other settings, one could extend the model to allow for time-varying loadings, but this more flexible version of the model would align with a different scientific question and come at the expense of decreased interpretability. We also assume that 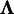 contains structural zeros such that each row of the loadings matrix contains only one non-zero element; this structure means that each observed outcome is a measurement of only a single latent factor. The decision to incorporate structural zeros in the loadings matrix is supported by behavioral science concepts (e.g., Positive and Negative Affect Schedule; Watson et al., 1988), which can be used to classify a given emotion as a measurement of a specific category of emotional state.

### 3.2 Structural submodel

The structural submodel captures the evolution of the latent factors, 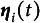 , over time. In the motivating data, these latent factors are psychological states (e.g., positive/negative affect, valence, arousal, etc.) assumed to generate the measured emotions. We use a multivariate OU process, which can be understood as a continuous-time analog of a VAR process and has the ability to capture temporal volatility. Here, we assume a bivariate OU process ( 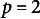 ) for illustrative purposes. The stochastic differential equation definition of the bivariate OU process is (2)

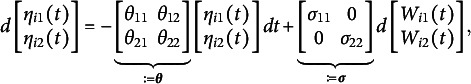

where the diagonal elements of matrix 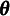 capture the mean-reverting tendency of the latent factors and the off-diagonal elements of 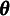 capture correlation between the latent factors. The diagonal elements of 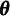 are required to be positive. Here, we assume the mean is 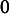 . Our motivating emotion data come from an observational mHealth study and we assume that emotions are in a steady state (i.e., the process is stationary). In a different setting in which a change over time might be expected, an analyst may want to use an alternative version of the OU process that has a time-dependent mean to capture time since the start of the study (i.e., an OU process that includes a term for time-varying drift), but we do not consider that here.

The matrix 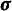 , with elements 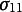 and 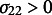 , describes the volatility of the process, where 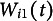 and 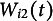 are both standard Brownian motion. In general, the standard definition of the OU process allows 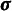 to take non-zero values in the off-diagonal. By restricting 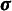 to be a simpler diagonal matrix here, we consider the Brownian motion terms as separate noise processes for each latent factor and thus capture all correlation between the latent factors through the 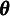 matrix. We also require that all eigenvalues of the 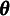 matrix have a positive real part; this constraint ensures a mean-reverting process (Tran et al., 2021a).

Although our dynamic factor model does not explicitly include a traditional random slope (e.g., 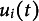 ), as commonly assumed in standard mixed models for the analysis of longitudinal data, our model does include a component that allows the change in latent factors to vary stochastically across time. In our model, this component is our structural submodel, the OU process. The stochastic differential equation definition of the OU process (Equation (2)) emphasizes the randomness in the change of the latent factors over time via the population-level volatility parameter 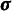 ; this randomness is analogous to the individual-specific random variation resulting from the population-level variance parameter of a random slope in a mixed model.

### 3.3 Likelihood definition

Rather than taking a Bayesian strategy or relying on the complete-data likelihood and taking an expectation-maximization (EM) approach to estimation, we directly maximize the likelihood of the observed data. Direct maximization of the marginal likelihood allows us to avoid repeatedly calculating values of the latent factors at each measurement occasion (via posterior sampling in a Bayesian framework or via complex integrals in the E-step of the EM algorithm). Thus, our approach is more scalable to the ILD setting.

In existing literature, the OU process is most often defined using its conditional distribution. If our *p* latent factors for individual *i*, denoted by vector 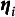 , follow an OU process, then the conditional distribution of the latent factors at time *t* given the previous value at time *s*, where 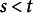 , is 

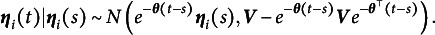



This distribution assumes that the initial value of the OU process is drawn from its stationary distribution, 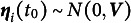 , where the stationary variance is 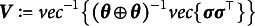 . Here, 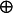 denotes the Kronecker sum, defined for square matrices 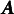 and 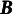 of sizes *a* and *b*, respectively, as 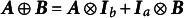 ; and the 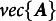 operation consists of stacking the columns of matrix 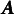 into a column vector.

The conditional distribution can be challenging to work with in the context of ILD, as it requires computing products sequentially across all measurement times within the likelihood. To simplify computation in our ILD setting, we integrate out the latent factors so that we can simply maximize the observed data log-likelihood. This marginal likelihood depends on the joint distribution of the latent factors. The joint distribution of 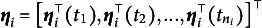 is 

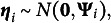

where 

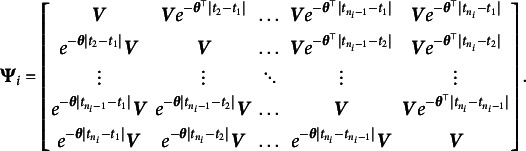



The dimension of the marginal OU covariance matrix 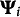 still scales with the number of longitudinal measurements and so to make our approach computationally amenable to the ILD setting, we take advantage of the fact that the OU process has the Markov property. As a result of this property, the inverse of the marginal covariance matrix—the precision matrix—is block tri-diagonal. Thus, it is much simpler to evaluate the likelihood for the OU process when written in terms of the sparse precision matrix, compared to either the dense marginal covariance matrix or as a product of many conditional distributions. As one of the key contribution of this article, we derive this sparse precision matrix: Let 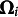 be the precision matrix of the OU process observed at 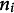 occasions. Then 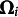 has the structure (3)

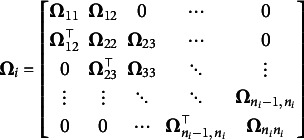

and each block indexed by *j* for 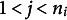 in the tri-diagonal matrix is (4)

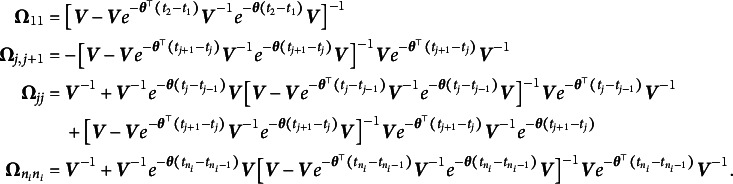



The derivation for each block is given in Section A.3 of the Supplementary Material. Later, during estimation, we leverage the sparse precision matrix to simplify computation. This sparsity becomes particularly advantageous as the number of individuals and observations per individual (e.g., EMAs per individual) in a dataset increases, and it is critical to the scalability of our model to the ILD setting.

Together, the measurement and structural submodels imply that the observed longitudinal outcomes are normally distributed with mean 0 and covariance 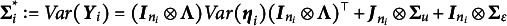 , where 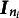 is an 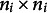 identity matrix and 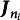 is an 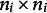 matrix of ones. We estimate the OUF model by minimizing the following function, which equal to twice the negative log-likelihood up to a constant: 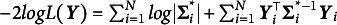 . As with other likelihood-based methods such as mixed effects models, our approach assumes that data are missing at random (MAR).

### 3.4 Identification issues

Before fitting our model, we must make additional assumptions to address identifiability issues common to factor models. Because both 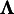 and 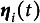 are unknown, multiplying 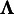 by some matrix, say 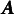 , and multiplying 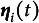 by 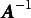 will result in the same model. To make a factor model identifiable, constraints must be placed on either the loadings matrix or the latent factors. Aguilar & West (2000) and Carvalho et al. (2008), for example, make the standard assumption of requiring the loadings matrix to be triangular while Tran et al. (2021b), for example, fix the variance of the latent factors at 1. The main disadvantage of assuming that 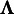 has a triangular structure is that the order of the longitudinal outcomes matters, and so the structure of this matrix is less intuitive to specify based on behavioral science literature. Assuming that the latent factors have a variance of 1 simply means that we model the latent psychological constructs on the correlation scale.

Thus, to make our model identifiable, we fix the scale of the latent factors but propose a novel approach for doing so. Letting 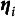 be the 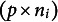 -length vector of latent variables values stacked over measurement occasions, we constrain 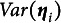 to have diagonal elements equal to 1. This constraint means that the OU process must have a stationary variance equal to 1. By fixing the scale of the latent factors, we can allow the elements of the loadings matrix 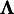 to vary almost freely during estimation. For a generic 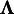 (without structural zeros), the only constraint on the loadings matrix is that the sign of the first element must be positive. Together these constraints make our model identifiable; the constraint on the OU process identifies the scale and the constraint on the first element of the loadings matrix identifies the direction. Because we later make the simplifying assumption that 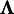 contains structural zeros with a single non-zero loading per row, flipping the signs on both the loadings and the latent factors results in the same model; we choose to keep the signs that correspond to the most relevant interpretation of the model given the application. Another constraint could be added to require that one loading per column of 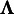 is positive; this would avoid sign flipping.

To impose this identifiability constraint, we use a set of *p* constants to re-scale the OU process parameters. We summarize this identifiability constraint for the bivariate ( 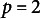 ) OU process as: using a pair of positive scalar constants 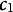 and 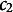 , we can re-scale an arbitrary OU process parameterized by 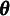 and 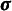 to have stationary variance of 1, where this re-scaled OU process is parameterized by 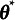 and 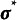 according to (5)

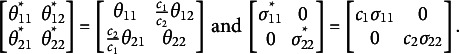



In Section A.4 of the Supplementary Material, we show why this re-scaling approach works for any mean-reverting OU process. This constraint can also be extended to OU processes of higher dimensions.

Although this identifiability assumption allows us to identify the magnitude of the loadings in the factor model, it does so only up to a sign change. Consider again the case of a bivariate OU process. To make this example more concrete, suppose also that one of the latent factors, 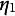 , is measured by the positive emotions and the other latent factor, 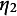 , is measured by the negative emotions collected in the motivating mHealth study. The likelihood for our model is equivalent for pairs of scaling constants 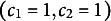 and 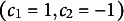 . In practice, the model would be the same under both pairs of scaling constants (and so we restrict 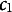 and 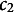 to be positive during estimation) but interpretation of model parameters would differ. After estimation, the signs of estimated model parameters can easily be flipped to match the most relevant interpretation of the data by multiplying estimates of 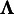 and 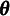 by a 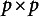 matrix with the constants along the diagonal. In this two-factor example, it would make sense to choose signs such that 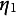 and 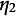 are negatively correlated and higher values of the latent factors correspond to higher values of the measured emotions. As a result, 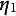 could be interpreted as representing positive affect and 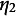 as negative affect, both of which are two traditional psychological constructs often used in behavioral science (Watson et al., 1988).

### 3.5 Estimation algorithm

To fit this model, we take an iterative approach to estimation in which we directly maximize the marginal likelihood of our observed longitudinal outcome using a block coordinate descent algorithm and rely on simpler existing models to inform the initial parameter estimates. To increase the computational efficiency of this estimation algorithm, we (i) take advantage of tractable analytic gradients for the measurement submodel, avoiding the need to calculate computationally expensive numerical gradients; (ii) leverage the Markov property of the OU process and use the computationally-simpler sparse precision matrix derived in Equation (3), rather than the dense covariance matrix; and (iii) implement the code used to repeatedly calculate these numerical gradients and the sparse precision matrix in C++, using R for the rest of our code.

In the block coordinate descent algorithm, we split parameters into two different blocks: one block for parameters in the measurement submodel ( 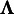 , 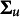 , 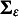 ) and the other for parameters in the structural submodel ( 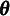 , 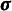 ). Note that each element of these blocks is actually a matrix of parameters. Within each block-wise iteration, we minimize the log-likelihood with respect to one block of parameters, given the current estimates of the other block of parameters, using Newton algorithms as implemented in R’s stats package (R Core Team, 2022). By updating parameters in blocks, we can leverage the availability of analytic gradients for parameters in the measurement submodel. The Kronecker structure of the covariance matrix for each individual’s longitudinal outcomes 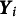 allows us to derive these analytic gradients. The gradient of the log-likelihood for a single individual with respect to one of the measurement submodel parameters, 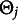 , has the general form (6)

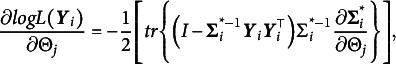

where the exact form of 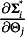 depends on the specific parameter; either 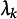 , 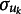 , or 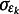 .

The complete set of analytic gradients is given in Section A.5 of the Supplementary Material. The computational advantage of using the analytic gradient, as opposed to a numerical approach to differentiation, is particularly notable as the number of longitudinal outcomes—and thus parameters in the measurement submodel—increases.

Prior to maximizing the marginal likelihood, we use a cross-sectional factor model to initialize 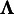 , 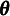 , and 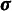 , and use linear mixed models to initialize 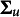 and 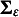 . Then, we iteratively update parameter estimates using the following block coordinate descent algorithm:

1. *Initialize estimates of* 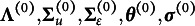 . *Measurement submodel parameters are always initialized empirically; for structural submodel parameters, two sets of initial estimates are considered—an empirical set of values estimated from cross-sectional factor scores and a default set of values. The set of values that corresponds to the higher log-likelihood given the current data is used.*
2. *Set iteration index* 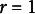 *and convergence indicator* 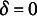 . While 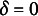 ,
  - *Update block 1 (measurement submodel):*


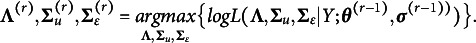


*Maximization is done via a Newton-type algorithm (nlm; R Core Team, 2022) using analytic gradients (Equation (6)).*
  - *Update block 2 (structural submodel):*


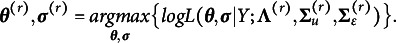


*Maximization is done via a quasi-Newton algorithm (nlminb; R Core Team, 2022) using numerical gradients and takes advantage of the sparsity of the OU precision matrix to increase the speed of this step. A large positive penalty is added to the negative log-likelihood within the optimization algorithm if a proposed* 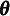 *does not have eigenvalues with positive real parts.*
  - *Using Equation (5), re-scale OU parameters to satisfy the identifiability constraint.*
  - *Check for block-wise convergence: Let* 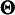 *be a vector containing all elements of* 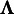 , 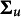 , 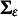 , 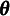 , *and* 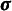 . *Then, calculate*


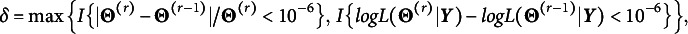


*where all operations on* 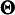 *are element-wise.*
  - *Update r*: 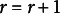

3. *Estimate Fisher Information-based standard errors from numerical approximations to the Hessian of the log-likelihood,* 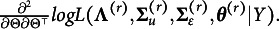

Note that when estimating standard errors, the parameterization of the likelihood differs slightly: the likelihood now depends on only one of the parameter matrices in the structural submodel, 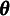 , and not the other, 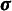 . This change in parameterization is a result of the identifiability constraint that is placed on the stationary variance of the OU process. Since we are no longer conditioning on fixed measurement submodel parameters in step 3, we restrict 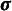 to be a function of 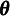 , where this function is derived from the identifiability constraint; thus, the likelihood is not over-parameterized. Standard error estimates for 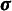 can be calculated quickly and easily using a parametric bootstrap. By sampling values of 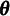 from a normal distribution defined by its point estimate and estimated covariance matrix, bootstrapped values of 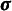 are calculated as a function of 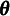 and a confidence interval can be estimated based on the empirical distribution. More details on the parameterization of the log-likelihood for standard error estimation are in Section A.6 of the Supplementary Material.

## 4 Simulation study

We conduct a simulation study to assess (i) the bias and variance of estimates when the OUF model is specified with the correct number of latent factors and (ii) the ability of Akaike information criterion (AIC) and Bayesian information criterion (BIC) to select the model with the correct number of latent factors among models with mis-specified numbers of latent factors and loadings matrices.

### 4.1 Data generation for assessing bias and variance

We assume that 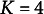 longitudinal outcomes (e.g., emotions) are recorded over time for 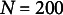 individuals. For individual *i*, these intensively measured longitudinal outcomes are recorded at 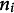 different occasions (e.g., EMAs) where 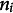 takes a random integer value between 10 and 20. The gap time between each measurement occasion is drawn from a 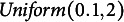 distribution, resulting in an average maximum follow-up time of about 14.25. Although our choice of four longitudinal outcomes is smaller than the number of outcomes often seen in ILD, we chose this number to balance between the complexity of our data and model, and the computational demands of a simulation study. To emphasize the importance of intensive measurements when fitting our model, we also illustrate model performance in a more challenging non-ILD setting in which longitudinal outcomes are measured less frequently. In this supplementary setting, 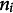 takes a random integer value between 2 and 7, where these measurement occasions are randomly distributed with uniform probability across a follow-up time of 14.25.

We consider simulated data in three different true parameter settings in which the bivariate OU process has varying degrees of autocorrelation (see Section A.7 of the Supplementary Material for exact values). Using each true OU process, we generate the observed longitudinal outcomes (for both the ILD and non-ILD) by drawing from 
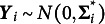
 where 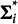 is defined using (7)

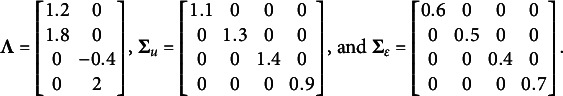



When fitting this model, we assume that the zeros within the loadings matrix, random intercept covariance matrix, and measurement error covariance matrix are known.

Importantly, some of the parameter values used to generate the data are different from the parameters that will be estimated by the model; this difference is a side effect of the identifiability assumption. While unbiased estimates of 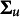 and 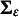 will match the values used in data generation, the values of 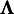 and the OU process parameters 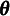 and 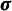 will differ. As a result of the re-scaling approach for identification, the estimated OU process has a stationary variance of 1. The additional variation present in the OU process during data generation must be absorbed by the loadings matrix 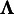 . Specifically, the data-generating loadings matrix will be re-scaled according to 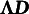 where 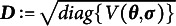 and 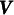 is the stationary variance of the OU process. 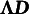 will be estimated by our algorithm. The data-generating OU parameters 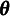 and 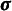 will be re-scaled according to scalar constants chosen such that the stationary variance of the re-scaled OU process is equal to 1 via Equation (5). True parameter values indicated in the simulation results have all been re-scaled to match the values targeted by our estimation algorithm. In setting 2, the true OU process used to generate data does have a stationary variance equal to 1 and thus the target parameter values do match the data-generating parameter values.

### 4.2 Bias and variance results

**Figure 2:**
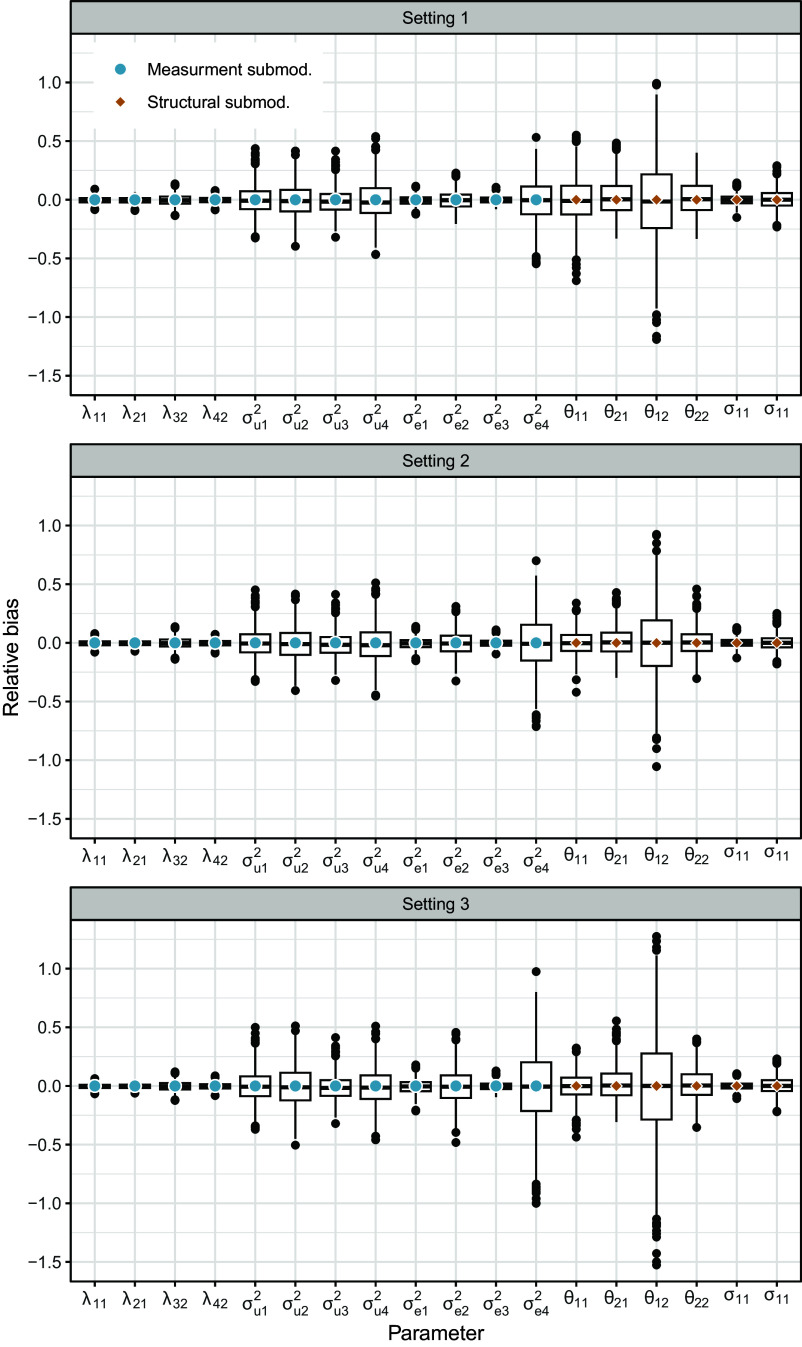
Results from ILD simulation study. Relative bias of parameter estimates from the block coordinate descent algorithm for the three different settings in which the true OU process differs. Relative bias is calculated as (estimate - truth) / truth and is summarized across the 1,000 simulated datasets with box plots. The colored dots indicate 0 bias.

**Figure 3:**
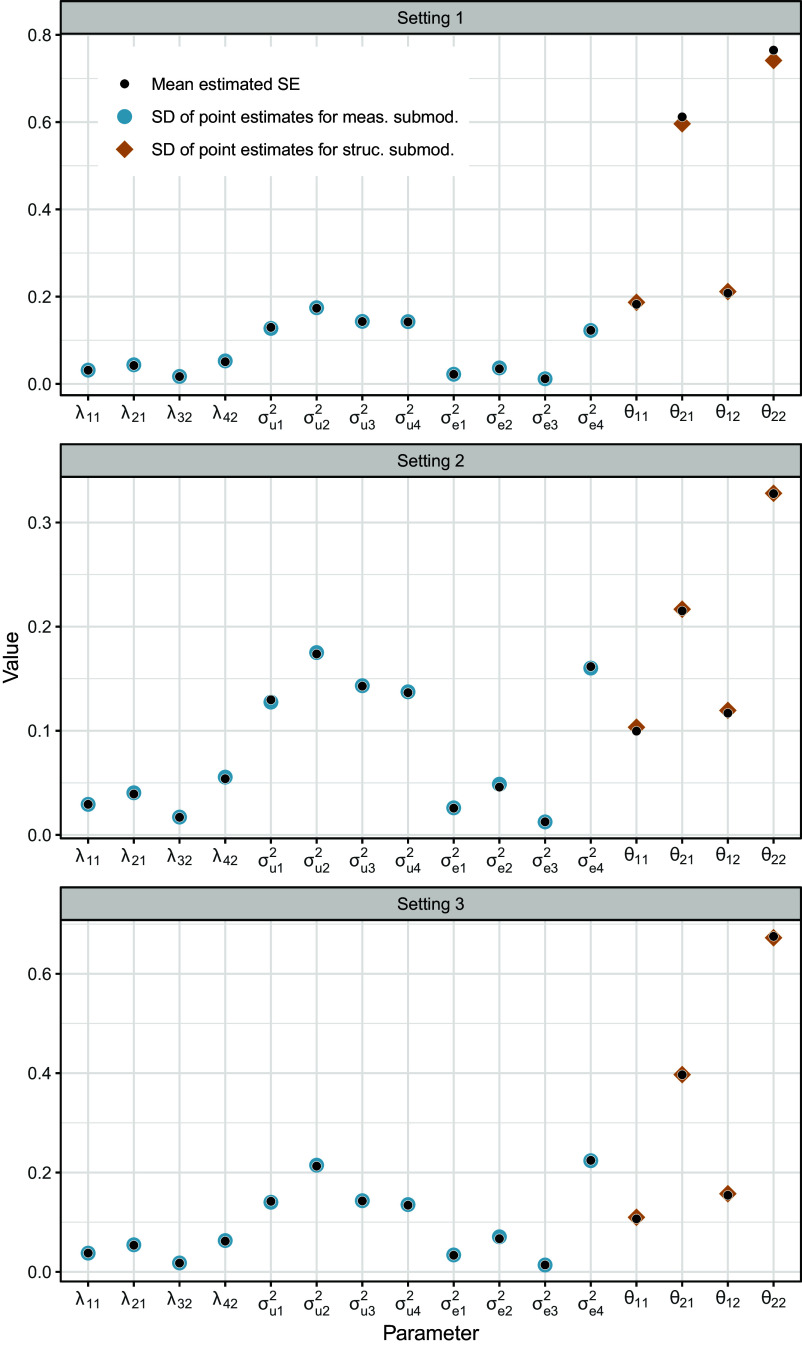
Results from ILD simulation study. Comparison of estimated standard errors (from Fisher information) and standard deviation of point estimates. The similarity of the standard error estimates and empirical standard deviation suggests that the standard errors are of appropriate size. Note that the standard error estimate for 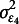 is missing for one dataset in setting 3 (see Section A.8 of the Supplementary Material for details).

In each of the three ILD settings and three non-ILD settings, we generate 1,000 datasets and carry out the estimation algorithm. We focus here on the results from the ILD settings. Relative bias for the final point estimates is shown in Figure 2 and information-based standard errors are summarized in Figure 3. Relative bias is calculated as (estimate - truth) / truth. In all settings, we consistently recover unbiased estimates of the true values and find that the averages of the standard errors are similar to the empirical standard deviations of the point estimates, indicating that confidence intervals will have close to nominal coverage. We provide more results tables in the Supplementary Material that summarize relative bias, root mean squared error, empirical standard deviations and estimated standard errors, and coverage rates; see Supplementary Tables 1–3. For one dataset, numerical issues result in a negative variance estimate; this specific case is discussed in Section A.8 of the Supplementary Material.

In the non-ILD settings, we also find that our method performs well for most parameters; however, relative bias is higher for the OU process parameter 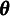 . 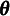 captures the correlated change in the latent factors over time, which requires measurements to be taken close enough together for the correlation to be captured. As the time between measurement occasions increases (i.e., as we go from ILD to non-ILD), capturing this correlation becomes more challenging and thus estimating 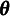 does too. Large true values of 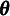 exacerbate this issue, as they correspond to settings in which correlation decays rapidly. Simulation results from the non-ILD settings, which illustrate this pattern, are presented in full in Section A.9 of the Supplementary Material.

### 4.3 Data generation for model selection

Because ILD consist of many different outcomes, determining the appropriate number of latent factors for summarizing these multiple outcomes may frequently be of interest. As such, we carry out a simulation study in which we evaluate the ability of AIC and BIC to correctly select the true model among the misspecified models. The formulas for AIC and BIC take into account our identifiability constraints. Letting 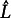 denote the maximized value of the marginal (observed data) likelihood of the OUF model; *q* be the total number of non-zero parameters in 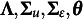 and 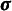 ; *p* be the number of latent factors (which corresponds to the number of scaling constants needed to impose the identifiability constraint); and *N* be the total number of independent individuals in the data, then AIC is calculated as 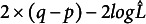 and BIC is calculated similarly as 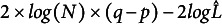 .

Assuming the same true measurement submodel parameters as before, we now generate ILD from five different factor models. Note that we do not consider non-ILD here. We set the true OU process parameters such that the data-generating OU processes vary in the level of correlation between the latent factors and thus in the amount of signal available to distinguish the latent factors from each other. We generate data from the following models: a one-factor model, a two-factor model with low signal (where the stationary correlation between 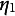 and 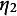 is 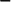 0.72), a two-factor model with high signal (where the stationary correlation between 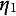 and 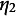 is 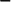 0.21), a three-factor model with low signal (where the stationary correlation between 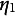 and 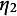 is 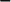 0.76, between 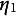 and 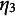 is 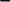 0.64, and between 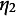 and 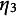 is 0.37), and a three-factor model with high signal (where the stationary correlation between 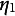 and 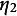 is 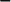 0.27, between 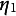 and 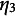 is 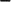 0.36, and between 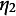 and 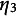 is 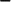 0.24).

The various structures of these data-generating models can be interpreted as representing different beliefs about underlying psychological states. Data-generating parameter values are given in the Section A.7 of the Supplementary Material. For 100 datasets generated from each of these true models, we fit a one-, two-, and three-factor model and compare information criteria. For fitted models with misspecified numbers of latent factors, the loadings matrix is also misspecified; for fitted models with the true number of latent factors, the structure of the loadings matrix is correctly specified. When fitting the models, we specify the structure of the loadings matrix as described in Section A.7 of the Supplementary Material. We do not consider a four-factor model in this simulation study because our data only contain four longitudinal outcomes and so fitting a four-factor model would no longer fall into the dimension-reduction setting that motivates this work.

### 4.4 Model selection results

We present model selection results in Table 1. In both the high and low signal settings, the model with the lowest AIC and BIC most often has the same number of factors as the true model used to generate the data. For models fit to data generated from a true model with three factors, BIC incorrectly selects a model with two factors more often than AIC. This difference make sense given the increased penalty that BIC places on model complexity. For ILD of this size ( 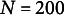 ), estimation becomes more difficult as the number of factors increases and so, for a few simulated datasets, our algorithm did not converge within the allotted maximum number of block-wise iterations (see Section A.8 of the Supplementary Material for details). Furthermore, we also noticed that when the incorrect number of latent factors is specified (and, as a result, the loadings matrix structure is misspecified), we find that convergence becomes slightly more challenging and additional iterations of the block coordinate descent algorithm are required.

**Table 1:** For datasets generated under each true model, we summarize the percent of times that the model-selection metric chose the fitted model with the indicated number of factors. When generating data from models with 2 and 3 factors, we considered two different settings: a high signal setting in which latent factors have lower correlation and a low signal setting in which latent factors have high correlation. The settings in which the fitted model has the same number of factors as the true data-generating model are emphasized with bold orange text. These results are presented for datasets on which the algorithm either converged or reached the maximum number of iterations (200) for all three models. See Section A.8 of the Supplementary Material for more details.

True Model		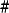 Factors in Fitted Model with Best AIC			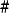 Factors in Fitted Model with Best BIC		
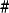 Factors	Signal	1	2	3	1	2	3
1	-	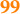	0	1	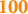	0	0
2	Low	0	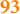	7	4	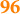	0
2	High	0	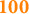	0	0	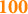	0
3	Low	0	0	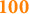	0	8	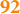
3	High	0	0	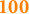	0	0	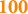

While AIC and BIC perform similarly, we recommend use of BIC in practice, as the increased penalty placed on model complexity aligns well with the dimension-reduction goal of factor models.

## 5 Application to mHealth emotion data

We use our method to analyze the data on momentary emotions collected in the mHealth study. We fit three different OUF models in which we summarize the longitudinal responses to 18 emotion-related questions as either one, two, or three latent factors. The measured emotions that we model are: happy, joyful, enthusiastic, active, calm, determined, grateful, proud, attentive, sad, scared, disgusted, angry, ashamed, guilty, irritable, lonely, and nervous.

Behavioral scientist have a variety of theories that describe how these measured emotions relate to underlying psychological states (e.g., Gilbert et al., 2008; McManus et al., 2019; Reich et al., 2003; Reisenzein, 1994; Remington et al., 2000; Robinson et al., 2020), and so we aim to compare the fit of models with different numbers of latent factors using this mHealth data. The one-factor OUF model assumes that positive and negative emotions are generated from a single common underlying factor (i.e., a single spectrum that ranges from positive to negative affect; Robinson et al., 2020). The two-factor OUF model assumes that the emotions are measurements of two distinct-but-correlated emotional states, which we interpret as positive affect and negative affect (Reich et al., 2003). In this model, happy, joyful, enthusiastic, active, calm, determined, grateful, proud, and attentive measure positive affect; and sad, scared, disgusted, angry, ashamed, guilty, irritable, lonely, and nervous measure negative affect. Finally, in the three-factor OUF model, we further divide the positive emotions into two latent factors that differ by the level of activation or arousal; we call these factors high arousal positive affect—measured by feeling grateful, proud, enthusiastic, active, determined, attentive—and no-to-low arousal positive affect—measured by feeling calm, happy, and joyful (Gilbert et al., 2008; McManus et al., 2019; Reisenzein, 1994; Remington et al., 2000). The negative emotions are still assumed to be generated from one latent factor. Due to the direct link between our model specification and behavioral science theories, our analysis approach enables comparison of the fits of these three models and allows us to investigate what level of dimension-reduction is appropriate for capturing the dynamics of the emotions measured in this mHealth study. At the same time, our model accounts for correlation between repeated measurements and related psychological states. This analysis highlights the advantages of the dynamic factor model over approaches such as PCA, which does not allow for direct incorporation of behavioral science theories into the model structure or consider the longitudinal aspect of the data, or flexible spline-based mixed models, which would be too computationally challenging to fit to all 18 outcomes simultaneously.

Specifying these three dynamic factor models and comparing their fits allows us to investigate what level of dimension-reduction is appropriate for capturing the temporal dynamics of the emotions measured in this mHealth study.

For comparison, we also take a more standard approach to analysis and fit mixed models with random slopes and random intercepts to each of the emotion outcomes separately. Ideally, we would fit a multivariate mixed model to all 18 outcomes simultaneously. However, fitting a model with a 36-dimensional covariance matrix for the random slope and random intercept is not practical. To compare the fit of the separate 18 mixed models to our dynamic factor models (with one, two, and three latent factors), we calculate a value of AIC based on the combined likelihood of the 18 fitted mixed models; we do the same for BIC. The resulting AIC and BIC values are worse than those for all three dynamic factor models (presented below). More detailed results for these mixed models are provided in Section C.3 of the Supplementary Material.

Of the three dynamic factor models considered, both AIC and BIC indicate that the two-factor model fits best: 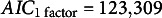 vs. 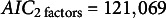 vs. 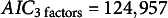 and 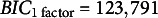 vs. 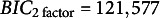 vs. 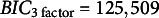 . Some psychological theories support our conclusion that two factors represent our data better than one as it suggests that positive and negative affect are not opposites, rather they capture distinct-but-correlated components of psychological state (Reich et al., 2003). The lower AIC and BIC of the two-factor model compared to the three-factor model suggest that the emotions corresponding to high arousal positive affect and no-to-low arousal positive affect are not different enough to justify the additional complexity of the three-factor model given the current data. The strong estimated correlation (0.995) between the latent factors for high arousal positive affect and no-to-low arousal positive affect further supports this conclusion.

**Figure 4:**
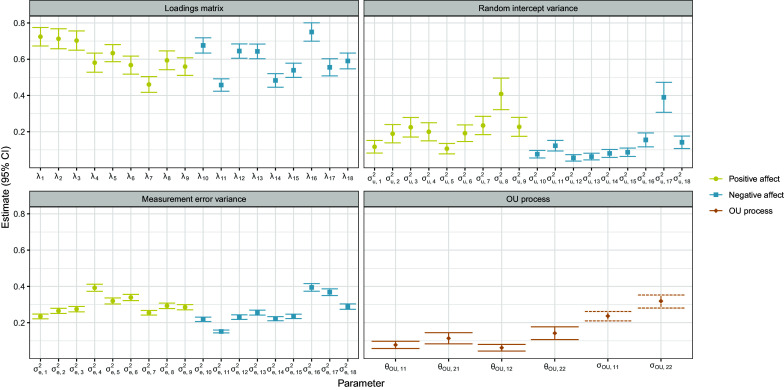
Point estimates and corresponding 95% confidence intervals (CI) for each of the parameter matrices in our two-factor OUF model. Intervals for OU parameters 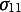 and 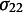 are based on a parametric bootstrap. Because we assume structural zeros in the loadings matrix are known, each emotion has only a single loading. Parameter subscripts 1-18 correspond to the emotions as follows: 1 = happy, 2 = joyful, 3 = enthusiastic, 4 = active, 5 = calm, 6 = determined, 7 = grateful, 8 = proud, 9 = attentive, 10 = sad, 11 = scared, 12 = disgusted, 13 = angry, 14 = ashamed, 15 = guilty, 16 = irritable, 17 = lonely, 18 = nervous.

**Figure 5:**
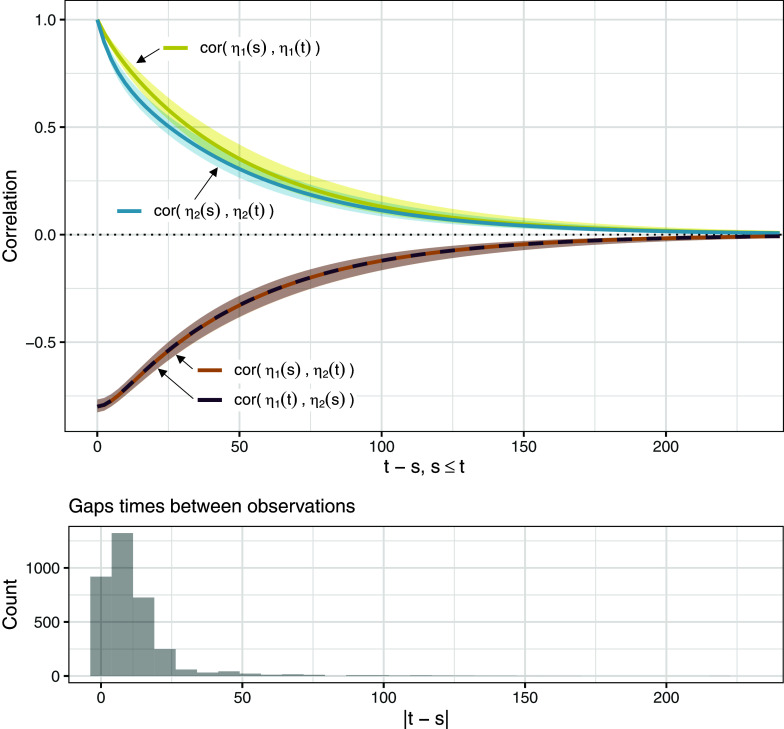
The top panel shows the decay in autocorrelation and cross-correlation between latent factors that represent positive affect ( 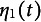 ) and negative affect ( 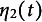 ) across increasing gap times, where time is measured in hours. Curves are calculated using OU parameters estimated from emotions measured in the mHealth study. The shaded bands indicate the 2.5th and 97.5th percentiles of a parametric bootstrap. The bottom plot summarizes the distribution of the observed gap times (in hours) between measurements for all individuals in the mHealth study.

For the bivariate OUF model, point estimates and 95% confidence intervals are in Figure 4. Coefficient estimates from the fitted one-factor and three-factor OUF models are given in Section B.1 of the Supplementary Material. For the two-factor model, measures of happiness, joy, and enthusiasm are most strongly correlated with positive affect and measures of sadness and irritability are most strongly correlated with negative affect. We can examine the OU process parameter estimates to gain insight into the latent dynamics of positive and negative affect. 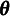 describes the rate at which the latent factors revert back toward the mean. Because the estimates of the diagonal elements of 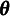 are similar, and the estimates of the off-diagonal elements of 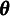 are similar, these results indicate that the latent factors for positive and negative affect have fairly symmetric dynamics. That is, the impact of a change in positive affect on negative affect is similar to that of negative affect on positive affect. Estimates of 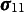 and 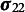 suggest that the dynamics of the latent factor for negative affect are slightly more volatile than those for positive affect. We can also use the estimated parameters of the OU process to understand the latent dynamics of positive and negative affect by plotting the degree of correlation for these two latent variables across varying time intervals between consecutive observations (see Figure 5). We see that positive and negative affect are negatively correlated as expected, and that the correlation between the latent states decays slowly. The similarity in the correlation curves highlights the symmetry between the estimated dynamics.

We can also examine the variance estimates for all components of our model—the latent factors, random intercepts, and error terms—in order to help understand potential sources of variability. The relatively high variance estimates for the random intercepts for pride and loneliness suggest that these two emotions have higher variability across participants and vary less within participants; this pattern is consistent across all three OUF models. To gain further insight into the role of state vs. trait within this set of emotions, we can calculate the proportion of total variance explained by the latent process vs. the random intercepts for the set of 18 emotions at a fixed time point. We find that the dynamic latent factors explain more of the variability in happy (69% from the latent factors vs. 15% from the random intercept) and disgusted (79% vs. 11%), for example. On the other hand, the random intercepts explain more variability in proud (30% from the latent factors vs. 35% from the random intercept) and lonely (28% from the latent factors vs. 36% from the random intercept). The remaining proportion of variance is attributed to measurement error.

## 6 Discussion

We developed an estimation method for a dynamic OUF model that combines a factor model to summarize multivariate observed longitudinal outcomes (e.g., emotions) as lower dimensional latent factors (e.g., psychological states) and an OU process to describe the temporal evolution of the latent factors in continuous time. By using the OU process, instead of a discrete time approach such as a VAR process, the model can be applied to irregularly-measured ILD commonly produced by mHealth studies. Importantly, to make the model suitable for the ILD setting, we (i) derive a close-form likelihood for the marginal distribution of the observed longitudinal outcome that integrates over latent variables, (ii) derive the sparse precision matrix for the multivariate OU process, and (iii) leverage a mix of analytic and numeric gradients in our block coordinate descent algorithm. Together, these methodological contributions enable us to use our model to study the short- and long-term dynamics of the intensity of momentary emotions using ILD from an mHealth study. Code for implementing this method, along with example simulated data, are available on Github at https://github.com/madelineabbott/OUF.

The comparison via AIC and BIC of our dynamic factor model and simple mixed effects models fit to the motivating mHealth data (Section 5) highlights the importance of capturing the correlation between related outcomes in a computationally feasible manner, which the dynamic factor model does by summarizing the outcomes as a smaller number of correlated latent factors. In this comparison, modeling the correlation outweighs any information lost due to the dimension-reduction aspect of the dynamic factor model.

A key aspect of our approach to dimension-reduction via a measurement submodel is that it allows the data to drive the relationship between the measured items and the latent factors (via the estimated loadings matrix), rather than requiring pre-computation of composite scores (e.g., average values of the positive and negative emotions) to represent the latent variables. Using pre-computed composite scores that avoid use of the measurement submodel would potentially allow for a more complex structural submodel that could account for individual-specific variations in the OU process parameters (see Oravecz et al., 2011). Use of these pre-computed scores, however, would not allow for investigation into which measured items are the strongest indicators of the dynamic latent factors.

Our derivation of the sparse precision matrix for the multivariate OU process, in particular, enables us to simplify computation and avoid the conditional distribution of the OU process, which is often used in practice but becomes computational costly in the ILD setting. Furthermore, the marginal log-likelihood used in our method makes it more amenable to comparing models using information criteria, such as AIC or BIC. The Bayesian approach for fitting a similar model developed in Tran et al. (2021a) uses the conditional likelihood, which has not been marginalized over the latent factors or the random intercepts. This conditional likelihood is quite convenient for Bayesian inference, but is less convenient for information criteria-based comparisons of models. Generally, the marginal likelihood is preferred when calculating information criteria; the use of conditional vs. marginal likelihoods for comparisons of factor models is discussed further in Merkle et al. (2019).

Through the marginal distribution of the multivariate OU process, we parameterize our likelihood in terms of the standard OU drift ( 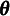 ) and volatility ( 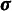 ) parameters. Having estimates for these parameters enables us to understand the dynamics of the latent factors, including generating new trajectories using the estimated values and examining the decay in the trajectories’ correlation over time. Through examination of decay in correlation over time, our method could help inform the design of future studies that aim to collect ILD by providing insight into how frequently the longitudinal outcomes must be measured in order to capture the correlation between them. In our simulation study for assessing bias and variance, we generated data under true OU processes that showed reasonably slow decay in correlation over time given the intervals between measurements in ILD. We found that estimation of the OU parameters is difficult if correlation decays quickly relative to gaps between measurements (i.e., in the non-ILD setting). When longitudinal outcomes are measured frequently enough that correlation between consecutive measurements is captured, our method consistently returns unbiased estimates of the OU process parameters.

In addition to understanding decay in correlation, we can use the OUF model to partition the variance of our observed outcome into contributions from different sources; specifically, the latent factors, random intercepts, and measurement error. Comparing the relative magnitude of these contributions allows us to gain insight into the importance of short-term variations within individuals and long-term differences across individuals. In the motivating mHealth data, these short-term variations are interpreted as emotional states and the long-term differences are interpreted as traits. The results of our analysis of these data suggest that it may be more important to measure certain emotions (e.g., happy and disgusted) frequently if understanding their dynamics is of interest, while other emotions (e.g., proud and lonely) may require less frequent measurements as they are more stable within an individual. EMAs often ask study participants to respond multiple times per day to numerous questions that assess their current state and context, and so understanding the optimal frequency at which to measure certain outcomes of interest could help reduce the burden on participants.

In other contexts (e.g., studies with longer follow-up periods), researchers may not be primarily interested in understanding which emotions to measure, but may be more interested in how the relationship between the measured emotions and the latent factors change over time. In that case, developing an extension of the dynamic factor model that allows loadings to vary as a function of time may be of interest. Del Negro & Otrok (2008) and Eraslan & Schröder (2023) present models with time-varying loadings that could inform such extensions. Regardless of the scientific question of interest, in certain situations, assuming that factor loadings are constant across both individuals and time could potentially lead to misleading conclusions if changes in measurement patterns are not captured in the loadings and instead captured as changes in latent variables (McNeish et al., 2021). Cross-classified factor models (i.e., factor models with individual- and time-specific loading matrices; Vogelsmeier et al., 2024) and latent Markov factor models (i.e., models that cluster measurement occasions and then fit cluster-specific factor models that may vary in both loadings structure and numbers of latent factors; Vogelsmeier et al., 2019) have been proposed to detect changes in the measurement model. Extending these techniques for use with the dynamic factor model could be useful, although complications resulting from the use of a continuous-time multivariate stochastic process model for the latent factors would have to be addressed.

Because we focus on the analysis of ILD on momentary emotions, behavioral science theories can be used to inform the placement of the structural zeros in the loadings matrix. In a different setting, the relationship between the longitudinal outcomes and the latent factors may be more difficult to specify based on existing domain-specific literature; in this case, extending this work to enable learning of the location of the structural zeros could be useful. An easy way to use the current method to gain insight into the structure of the loadings matrix would be to use AIC or BIC to compare models that are constant in the number of latent factors but differ in the locations of the structural zeros.

While we assume that the measured items are continuous outcomes, this assumption may not be plausible in all settings, particularly if responses to items are highly skewed or zero-inflated. In Tran et al. (2021a), the authors propose a similar model that can also account for a mix of binary and ordinal items. They take a Bayesian approach to fitting their model. Our maximum likelihood-based approach could likely also be adapted, although computation time would increase due to increased model complexity.

Our model currently assumes that data are MAR; however, given that the emotions are self-reported, this assumption may not hold in practice as individuals may be less willing to respond to EMAs in certain emotional states. For example, closer examination of the motivating mHealth data finds that day-level average emotions do differ slightly between the days on which participants respond to 1–2 EMAs vs. days on which participants respond to 3–4 EMAs. Specifically, we find that positive emotions tend to be slightly higher and negative emotions tend to be slightly lower on days during which participants respond to 3–4 EMAs. Although this pattern is consistent across all emotions, the magnitude of the difference is small (e.g., the average difference in day-level average responses is 0.07 for positive emotions and 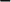 0.09 for negative emotions, where these differences on the scale of five-point Likert scale responses). Additionally, in many mHealth studies, individuals tend to respond to EMAs less frequently over time due to the gradually accumulating burden. In the motivating mHealth study, the number of EMA responses does decline slightly over the course of the study, with individuals responding to an average of 1.8 EMAs per day across the first five days of the study and an average of 1.5 EMAs per day across the final five days of the study. Furthermore, the missingness mechanisms for these individuals may be different from those for individuals who respond intermittently due to certain emotional states. Thus, considering methods for modeling various missingness mechanisms—including informative missingness—is an important and useful direction for future work. Recently, methods aimed at addressing the issue of non-ignorable missingness in ILD have been proposed. These methods use a variety of strategies, including selection models (which model the probability of response) and shared parameter models (which use shared latent variables to capture the likelihood of response). Cursio et al. (2019) take the latter approach and propose a latent trait shared-parameter mixed model in which a continuous latent factor, representing an individual’s likelihood of responding to an EMA, is shared between a mixed model for the outcome of interest and an item response theory model for the response status. On the other hand, Yuan et al. (2020) and McNeish (2025) take the former strategy and propose selection model-based approaches to account for informative missingness, emphasizing the suitability of these methods for ILD.

Although we use the sparse OU precision matrix, leverage the availability of analytic gradients for the measurement submodel parameters, and implement a portion of our algorithm in C++, the computation time of our estimation algorithm increases rapidly as the number of longitudinal outcomes increases. We successfully fit our model to a dataset containing 18 longitudinal outcomes but this does require approximately 27 hours. In order to make application of our model to datasets with larger numbers of longitudinal outcomes feasible, computational efficiency must be increased. Improving computational efficiency would be particularly advantageous in settings in which assessing measurement invariance is of interest, as this process requires repeatedly fitting models to different subgroups in the data. However, our proposed marginal likelihood-based method does have substantial computational benefits when compared to alternative methods. In comparison to the Bayesian approach proposed for fitting a similar model in Tran et al. (2021a), our approach requires less computation time. In a simulation study with *K* = 4 continous longitudinal outcomes measured at 10–20 occasions on *N* = 200 individuals, we found that estimation via our block coordinate descent algorithm required approximately 5% of the time required by the Bayesian approach proposed in Tran et al. (2021a) given the same computing resources. A comparison of point estimates from the two methods showed similar results, although our approach may lead to slightly more precise estimates of the OU process parameter 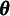 , particularly when computation time is limited. More details on this comparison are given in Section C.2 of the Supplementary Material.

In the simulation study and real data analysis presented in this work, we fit OUF models with one, two, or three latent factors, but the methods presented here extend to models with larger numbers of latent factors. Tran et al. (2021a) also focus on models with two or three latent factors. To fit their model, they derive an algebraic constraint on the 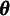 matrix requiring that 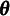 has eigenvalues with positive real parts; this constraint results in a latent process that is mean-reverting but possibly oscillating. The authors acknowledge that a limitation of this approach is that this constraint may not be easy to derive for a larger number of latent factors. In our work, we follow the eigenvalue constraint recommended in Tran et al. (2021a) but implement this constraint by adding a penalty to the likelihood. This penalty-based approach only requires us to calculate the eigenvalues of the 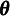 matrix, rather than derive an algebraic solution, and thus it is straightforward to increase the number of factors in our model.

Finally, the mHealth dataset to which we applied our method comes from a smoking cessation study and also contains information on demographic characteristics and on the timing of cigarette use. Including time-invariant baseline covariates (e.g., demographic variables) in the measurement would be a useful extension. Furthermore, the dynamic factor model could be extended to include a hierarchical OU process (i.e., one with individual-specific 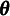 and/or 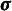 parameters) in order to account for heterogeneity among individuals attempting to quit smoking. A different stochastic process altogether (e.g., another process from the Matérn covariance class) could also be used. In our case, we treat the emotion items as a continuous outcome, but a version of the model proposed in Tran et al. (2021a) could be used to account for non-normal outcomes (e.g., binary or categorical outcomes). In behavioral science, specific emotional states, such as negative affect or craving, are expected to be correlated with cigarette use and so future work could involve combining our OUF model with a submodel for event-time outcomes. Rather than assuming a stationary process that has a constant mean, our model could also be modified to account for treatment or for drift in the OU process to better capture the dynamics of the latent processes after a key event.

## Supporting information

Abbott et al. supplementary material Abbott et al. supplementary material

## Data Availability

The mobile health data motivating this work are available upon reasonable request to the authors.

## Supplementary material

The supplementary material for this article can be found at https://doi.org/10.1017/psy.2025.10023. Example code and simulated data are available on Github at https://github.com/madelineabbott/OUF.
