# Supplementary material for: A Continuous-Time Dynamic Factor Model for Intensive Longitudinal Data Arising from Mobile Health Studies
Source: Psychometrika. 2025 Jun 16;90(3):1175–96. doi: 10.1017/psy.2025.10023 (PMC12439307; doi:10.1017/psy.2025.10023)
Supplement: Abbott et al. supplementary material [file S0033312325100239sup001.pdf]

Supplementary Material for:

A Continuous-Time Dynamic Factor Model for Intensive Longitudinal Data Arising from Mobile Health Studies

by

Madeline R. Abbott, Walter H. Dempsey, Inbal Nahum-Shani, Cho Y. Lam, David W. Wetter, and Jeremy M. G. Taylor

Section A

A.1 Derivation of the analytic form of the conditional covariance function of the OU process

Assume $\eta(t)$ is a p-dimensional Ornstein-Uhlenbeck (OU) stochastic process with a marginal mean of 0. From Vatiwutipong and Phewchean (2019), if we assume that the initial state $\eta(t_0 = 0)$ is known, then the cross-covariance function of the OU process at times s and t is

$$Cov\{\eta(s), \eta(t)|\eta(t_0 = 0)\} = \int_0^{\min(s,t)} e^{-\theta(s-u)}\sigma\sigma^\top e^{-\theta^\top(t-u)}du$$

where e^A is the matrix exponential. Note that we can assume that $t_0 = 0$ without loss of generality because this stochastic process is stationary. Using the identity for matrices A, B, and C that $vec(ABC) = (C^\top \otimes A)vec(B)$, we can re-write the vectorized version of the cross-covariance function as

$$vec\{Cov\{\eta(s), \eta(t)|\eta(t_0)\}\} = \int_0^{\min(s,t)} e^{-\theta(t-u)} \otimes e^{-\theta(s-u)}\ du\ vec\{\sigma\sigma^\top\}$$

We can also use the identity that $e^A \otimes e^B = e^{A\oplus B}$, so

$$vec\{Cov\{\eta(s), \eta(t)|\eta(t_0)\}\} = \int_0^{\min(s,t)} e^{[-\theta(t-u)]\oplus[-\theta(s-u)]}du\ vec\{\sigma\sigma^\top\} \tag{1}$$

Next, we simplify Equation 1 by pulling all the u's into a single term. For now, focus on

the term in the exponential:

$$\begin{aligned}[-\theta(t-u)] \oplus [-\theta(s-u)] &\overset{(a)}{=} -\theta(t-u) \otimes I + I \otimes (-\theta(s-u)) \\ &= -t(\theta \otimes I) + u(\theta \otimes I + I \otimes \theta) - s(I \otimes \theta) \\ &= -(t\theta \oplus s\theta) + u(\theta \oplus \theta)\end{aligned}$$

where equality (a) is by the definition of the Kronecker sum; $A \oplus B = A \otimes I_B + I_A \otimes A$, where I_A and I_B are identity matrices with dimensions of A and B, respectively. Now, substituting this new term back into the exponential term in Equation 1, we get

$$e^{[-\theta(t-u)]\oplus[-\theta(s-u)]} = e^{-(t\theta\oplus s\theta)+u(\theta\oplus\theta)} \tag{2}$$

We can simplify this further using the identity $e^{A+B} = e^A e^B$ if A and B commute. Letting $A = (t\theta) \oplus (s\theta)$ and $B = (\theta \oplus \theta)$, we first show that these terms commute:

$$\begin{aligned}A \cdot B &= [(t\theta) \oplus (s\theta)] \cdot [\theta \oplus \theta] \\ &= [t\theta \otimes I + I \otimes s\theta] \cdot [\theta \otimes I + I \otimes \theta] \\ &= (t\theta \otimes I)(\theta \otimes I) + (t\theta \otimes I)(I \otimes \theta) + (I \otimes s\theta)(\theta \otimes I) + (I \otimes s\theta)(I \otimes \theta) \\ &= (t\theta \otimes I)(\theta \otimes I) + (I \otimes \theta)(t\theta \otimes I) + (\theta \otimes I)(I \otimes s\theta) + (I \otimes s\theta)(I \otimes \theta) \\ &= (\theta \otimes I)\left[(t\theta \otimes I) + (I \otimes s\theta)\right] + (I \otimes \theta)\left[(t\theta \otimes I) + (I \otimes s\theta)\right] \\ &= [(\theta \otimes I) + (I \otimes \theta)] \cdot [(t\theta \otimes I) + (I \otimes s\theta)] \\ &= [(\theta \oplus \theta)] \cdot [(t\theta \oplus s\theta)]\end{aligned}$$

where line 4 uses the mixed-product property of the Kronecker product.
Referring back to Equation 2, we now have

$$e^{-(t\theta\oplus s\theta)+u(\theta\oplus\theta)} = e^{-(t\theta\oplus s\theta)} e^{u(\theta\oplus\theta)}$$

We can substitute this term into Equation 1 to get

$$\begin{aligned}vec\{Cov\{\eta(s), \eta(t)|\eta(t_0 = 0)\}\} &= \int_0^{\min(s,t)} e^{-(t\theta\oplus s\theta)} e^{u(\theta\oplus\theta)} du \; vec\{\sigma\sigma^\top\} \\ &= \int_0^{\min(s,t)} e^{u(\theta\oplus\theta)} du \; e^{-(t\theta\oplus s\theta)} vec\{\sigma\sigma^\top\}\end{aligned}$$

Now that we have rewritten the conditional cross-covariance function in this form, the only term that we need to integrate is $e^{u(\theta\oplus\theta)}$. We find

$$\int_0^{min(s,t)} e^{u(\theta\oplus\theta)} du = (\theta \oplus \theta)^{-1}\left[e^{min(s,t)(\theta\oplus\theta)} - I\right]$$

We now have an integral-free analytic form of the conditional cross-covariance function:

$$vec\{Cov\{\eta(s), \eta(t)|\eta(t_0 = 0)\}\} = (\theta \oplus \theta)^{-1}\left[e^{min(s,t)(\theta\oplus\theta)} - I\right] e^{-(t\theta\oplus s\theta)} vec\{\sigma\sigma^\top\}$$

Note that if $s = t$, then the conditional cross-covariance function simplifies to the conditional covariance function given in Vatiwutipong and Phewchean (2019).

A.2 Derivation of the analytic form of the marginal covariance function of the OU process

The analytic form of the conditional covariance function, given in Section 3.3 of the main paper, is based on the assumption that the initial state $\eta(t_0)$, with $t_0 = 0$ is *known*. We now derive the analytic form of the unconditional cross-covariance function that accounts for the additional uncertainty of an unknown initial state. From Vatiwutipong and Phewchean (2019), if $\eta(t_0)$, with $t_0 = 0$, is known, then

$$\mathbb{E}\{\eta(t)|\eta(t_0)\} = e^{-\theta t}\eta(t_0)$$

Assuming that $s \leq t$, from Lemma 1, we have

$$Cov\{\eta(s), \eta(t)|\eta(t_0)\} = vec^{-1}\Big\{(\theta \oplus \theta)^{-1}\Big[e^{(\theta\oplus\theta)s} - I\Big]e^{-(\theta t\oplus\theta s)}vec\{\sigma\sigma^\top\}\Big\}$$

If $\eta(t_0)$ is *unknown* and $t_0 = 0$, then using the Law of Total Covariance we can calculate

$$\begin{aligned}
Cov\{\eta(s), \eta(t)\} &= \mathbb{E}\big\{Cov\big(\eta(s), \eta(t)|\eta(t_0)\big)\big\} + Cov\big\{\mathbb{E}\big(\eta(s)|\eta(t_0)\big), \mathbb{E}\big(\eta(t)|\eta(t_0)\big)\big\} \\
&= vec^{-1}\Big\{(\theta \oplus \theta)^{-1}\Big[e^{(\theta\oplus\theta)s} - I\Big]e^{-(\theta t\oplus\theta s)}vec\{\sigma\sigma^\top\}\Big\} \\
&\quad + Cov\big\{e^{-\theta s}\eta(t_0), e^{-\theta t}\eta(t_0)\big\} \\
&= vec^{-1}\Big\{(\theta \oplus \theta)^{-1}\Big[e^{(\theta\oplus\theta)s} - I\Big]e^{-(\theta t\oplus\theta s)}vec\{\sigma\sigma^\top\}\Big\} \\
&\quad + e^{-\theta s}Var\big\{\eta(t_0)\big\}[e^{-\theta t}]^\top
\end{aligned}$$

If we assume that $\eta(t_0)$ is drawn from the stationary distribution, then $Var(\eta(t_0)) = vec^{-1}\big\{(\theta \oplus \theta)^{-1}vec\{\sigma\sigma^\top\}\big\}$. Then, we have

$$\begin{aligned}
Cov\{\eta(s), \eta(t)\} =& vec^{-1}\Big\{(\theta \oplus \theta)^{-1}\Big[e^{(\theta\oplus\theta)s} - I\Big]e^{-(\theta t\oplus\theta s)}vec\{\sigma\sigma^\top\}\Big\} \\
&+ e^{-\theta s}vec^{-1}\big\{(\theta \oplus \theta)^{-1}vec\{\sigma\sigma^\top\}\big\}[e^{-\theta t}]^\top
\end{aligned}$$

Now we simplify this function. Consider the terms involving θ in the first term of the sum,

$$(\theta \oplus \theta)^{-1}\Big[e^{(\theta\oplus\theta)s} - I\Big]e^{-(\theta t\oplus\theta s)}$$

We can simplify this expression using the fact that $e^A e^B = e^B e^A$ in our setting. This property means that both

$$(\theta \oplus \theta)^{-1}\big[e^{s(\theta\oplus\theta)} - I\big]e^{-(t\theta\oplus s\theta)} = e^{-(t\theta\oplus s\theta)}(\theta \oplus \theta)^{-1}\big[e^{s(\theta\oplus\theta)} - I\big] \tag{3}$$

and

$$(\theta \oplus \theta)^{-1}\left[e^{s(\theta\oplus\theta)} - I\right]e^{-(t\theta\oplus s\theta)} = (\theta \oplus \theta)^{-1}e^{-(t\theta\oplus s\theta)}\left[e^{s(\theta\oplus\theta)} - I\right] \tag{4}$$

Setting Equations 3 and 4 equal and cancelling the final term implies that

$$e^{-(t\theta\oplus s\theta)}(\theta \oplus \theta)^{-1} = (\theta \oplus \theta)^{-1}e^{-(t\theta\oplus s\theta)}$$

We will use this proof of the commutative property later and now return to our expression for the unconditional cross-covariance function, $Cov\{\eta(s), \eta(t)\}$,

$$\begin{aligned} Cov\{\eta(s), \eta(t)\} =& vec^{-1}\Big\{(\theta \oplus \theta)^{-1}\Big[e^{(\theta\oplus\theta)s} - I\Big]e^{-(\theta t\oplus\theta s)}vec\{\sigma\sigma^\top\}\Big\} \\ &+ e^{-\theta s}vec^{-1}\big\{(\theta \oplus \theta)^{-1}vec\{\sigma\sigma^\top\}\big\}[e^{-\theta t}]^\top \end{aligned} \tag{5}$$

Consider the second term in the sum,

$$e^{-\theta s}vec^{-1}\big\{(\theta \oplus \theta)^{-1}vec\{\sigma\sigma^\top\}\big\}[e^{-\theta t}]^\top$$

By applying the identity $vec(ABC) = (C^\top \otimes A)vec(B)$, we can rewrite the vectorized form of the expression as

$$\begin{aligned} vec\big\{e^{-\theta s}vec^{-1}\big\{(\theta \oplus \theta)^{-1}vec\{\sigma\sigma^\top\}\big\}[e^{-\theta t}]^\top\big\} &= e^{-\theta t} \otimes e^{-\theta s}vec\big\{vec^{-1}\big\{(\theta \oplus \theta)^{-1}vec\{\sigma\sigma^\top\}\big\}\big\} \\ &= e^{-\theta t} \otimes e^{-\theta s}(\theta \oplus \theta)^{-1}vec\{\sigma\sigma^\top\} \\ &= e^{-(\theta t\oplus\theta s)}(\theta \oplus \theta)^{-1}vec\{\sigma\sigma^\top\} \end{aligned}$$

Reversing the vectorization operation and applying the commutative property, we then get

$$\begin{aligned} e^{-\theta s}vec^{-1}\big\{(\theta \oplus \theta)^{-1}vec\{\sigma\sigma^\top\}\big\}[e^{-\theta t}]^\top &= vec^{-1}\big\{e^{-(\theta t\oplus\theta s)}(\theta \oplus \theta)^{-1}vec\{\sigma\sigma^\top\}\big\} \\ &= vec^{-1}\big\{(\theta \oplus \theta)^{-1}e^{-(\theta t\oplus\theta s)}vec\{\sigma\sigma^\top\}\big\} \end{aligned}$$

Plugging the term above into the second term of Equation 5, the cross-covariance function becomes

$$\begin{aligned} Cov\{\eta(s), \eta(t)\} &= vec^{-1}\Big\{(\theta \oplus \theta)^{-1}\Big[e^{(\theta\oplus\theta)s}e^{-(\theta t\oplus\theta s)} - e^{-(\theta t\oplus\theta s)}\Big]vec\{\sigma\sigma^\top\}\Big\} \\ &\quad + vec^{-1}\Big\{(\theta \oplus \theta)^{-1}e^{-(\theta t\oplus\theta s)}vec\{\sigma\sigma^\top\}\Big\} \\ &= vec^{-1}\Big\{(\theta \oplus \theta)^{-1}e^{(\theta\oplus\theta)s}e^{-(\theta t\oplus\theta s)}vec\{\sigma\sigma^\top\} - (\theta \oplus \theta)^{-1}e^{-(\theta t\oplus\theta s)}vec\{\sigma\sigma^\top\}\Big\} \\ &\quad + vec^{-1}\Big\{(\theta \oplus \theta)^{-1}e^{-(\theta t\oplus\theta s)}vec\{\sigma\sigma^\top\}\Big\} \\ &= vec^{-1}\Big\{(\theta \oplus \theta)^{-1}\big[e^{(\theta\oplus\theta)s}e^{-(\theta t\oplus\theta s)}\big]vec\{\sigma\sigma^\top\}\Big\} \\ &= vec^{-1}\Big\{(\theta \oplus \theta)^{-1}\big[e^{(\theta\oplus\theta)s-(\theta t\oplus\theta s)}\big]vec\{\sigma\sigma^\top\}\Big\} \end{aligned} \tag{6}$$

Equation 6 is the marginal cross-covariance function of the OU process when the initial state at time $t_0 = 0$ is *unknown*.

A.3 Derivation of the precision matrix for the OU process

We derive the sparse precision matrix for the multivariate OU process assuming an unknown initial state. This sparsity results from the Markov property. We use Ω to represent the precision matrix and Ψ for the covariance matrix.

First, we start in the simplest setting in which we assume a stationary univariate OU process with evenly spaced measurement occasions. The spacing of the measurement times is given by $|t_j - t_{j-1}| =: d > 0$. The covariance matrix takes the form,

$$\Psi = \frac{\sigma^2}{2\theta} \begin{bmatrix} 1 & e^{-\theta d} & \dots & e^{-\theta(n-2)\cdot d} & e^{-\theta(n-1)\cdot d} \\ e^{-\theta d} & 1 & \dots & e^{-\theta(n-3)\cdot d} & e^{-\theta(n-2)\cdot d} \\ \vdots & \vdots & \ddots & \vdots & \vdots \\ e^{-\theta(n-2)\cdot d} & e^{-\theta(n-3)\cdot d} & \dots & 1 & e^{-\theta d} \\ e^{-\theta(n-1)\cdot d} & e^{-\theta(n-2)\cdot d} & \dots & e^{-\theta d} & 1 \end{bmatrix}$$

We know that the univariate OU process is equal to the AR(1) process when measurements are evenly spaced, so the OU process precision matrix (assuming evenly spaced measurements) can be expressed as

$$\Omega = \frac{2\theta}{\sigma^2} \frac{1}{1 - e^{-2\theta d}} \begin{bmatrix} 1 & -e^{-\theta d} & \dots & 0 & 0 \\ -e^{-\theta d} & 1 + e^{-2\theta d} & \dots & 0 & 0 \\ \vdots & \vdots & \ddots & \vdots & \vdots \\ 0 & 0 & \dots & 1 + e^{2\theta d} & -e^{-\theta d} \\ 0 & 0 & \dots & -e^{-\theta d} & 1 \end{bmatrix}$$

Now, consider a more general setting in which measurements do not necessarily occur at evenly spaced intervals. Assume that $t_1 < t_2 < \cdots < t_{n-1} < t_n$. Then, the covariance matrix takes the form

$$\Psi = \frac{\sigma^2}{2\theta} \begin{bmatrix} 1 & e^{-\theta|t_2 - t_1|} & \dots & e^{-\theta|t_{n-1} - t_1|} & e^{-\theta|t_n - t_1|} \\ e^{-\theta|t_2 - t_1|} & 1 & \dots & e^{-\theta|t_{n-1} - t_2|} & e^{-\theta|t_n - t_2|} \\ \vdots & \vdots & \ddots & \vdots & \vdots \\ e^{-\theta|t_{n-1} - t_1|} & e^{-\theta|t_{n-1} - t_2|} & \dots & 1 & e^{-\theta|t_{n-1} - t_n|} \\ e^{-\theta|t_n - t_1|} & e^{-\theta|t_n - t_2|} & \dots & e^{-\theta|t_n - t_{n-1}|} & 1 \end{bmatrix}$$

and the precision matrix can be expressed as

$$\Omega = \frac{2\theta}{\sigma^2} \begin{bmatrix} \frac{1}{1-e^{-2\theta|t_2-t_1|}} & -\frac{e^{-\theta|t_2-t_1|}}{1-e^{-2\theta|t_2-t_1|}} & \dots & 0 & 0 \\ -\frac{e^{-\theta|t_2-t_1|}}{1-e^{-2\theta|t_2-t_1|}} & \frac{1-e^{-2\theta|t_3-t_1|}}{(1-e^{-2\theta|t_2-t_1|})(1-e^{-2\theta|t_3-t_2|})} & \dots & 0 & 0 \\ \vdots & \vdots & \ddots & \vdots & \vdots \\ 0 & 0 & \dots & \frac{1-e^{-2\theta|t_n-t_{n-2}|}}{(1-e^{-2\theta|t_2-t_1|})(1-e^{-2\theta|t_3-t_2|})} & -\frac{e^{-2\theta|t_n-t_{n-1}|}}{1-e^{-2\theta|t_n-t_{n-1}|}} \\ 0 & 0 & \dots & -\frac{e^{-2\theta|t_n-t_{n-1}}}{1-e^{-2\theta|t_n-t_{n-1}|}} & \frac{1}{1-e^{-2\theta|t_n-t_{n-1}|}} \end{bmatrix}$$

Next, we move from the one-dimensional case to the two-dimensional case. We start by

re-arranging the terms in the definition of the cross-covariance function for the bivariate OU process.

$$\begin{aligned}
Cov\{\eta(s),\eta(t)\} &= vec^{-1}\big\{(\theta\oplus\theta)^{-1}e^{s\wedge t(\theta\oplus\theta)-(\theta t)\oplus(\theta s)}vec(\sigma\sigma^\top)\big\}\\
&\overset{(a)}{=} vec^{-1}\big\{e^{s\wedge t(\theta\oplus\theta)}e^{-(\theta t)\oplus(\theta s)}(\theta\oplus\theta)^{-1}vec(\sigma\sigma^\top)\big\}\\
&= vec^{-1}\big\{\big[e^{s\wedge t\theta}\otimes e^{s\wedge t\theta}\big]\big[e^{-\theta t}\otimes e^{-\theta s}\big](\theta\oplus\theta)^{-1}vec(\sigma\sigma^\top)\big\}\\
&= vec^{-1}\big\{\big[e^{s\wedge t\theta}e^{-\theta t}\big]\otimes\big[e^{s\wedge t\theta}e^{-\theta s}\big](\theta\oplus\theta)^{-1}vec(\sigma\sigma^\top)\big\}\\
&= vec^{-1}\big\{\big[e^{-\theta(t-s\wedge t)}\big]\otimes\big[e^{-\theta(s-s\wedge t)}\big](\theta\oplus\theta)^{-1}vec(\sigma\sigma^\top)\big\}\\
&\overset{(b)}{=} vec^{-1}\big\{\big[e^{-\theta(t-s\wedge t)}\big]\otimes I(\theta\oplus\theta)^{-1}vec(\sigma\sigma^\top)\big\}\\
&= vec^{-1}\big\{(\theta\oplus\theta)^{-1}vec(\sigma\sigma^\top)\big\}e^{-\theta^\top|t-s|}\\
&:= V\cdot e^{-\theta^\top|t-s|}
\end{aligned}$$

where equality (a) is because these terms commute and equality (b) holds when we assume that $min(s,t)=s$. We can make this assumption without loss of generality because the matrices are symmetric. When $min(s,t)=t$, $Cov\{\eta(s),\eta(t)\}=e^{-\theta|t-s|}V^\top=e^{-\theta|t-s|}V$. Then, the covariance matrix is given by

$$\Psi=\begin{bmatrix}
V & Ve^{-\theta^\top|t_2-t_1|} & \dots & Ve^{-\theta^\top|t_{n-1}-t_1|} & Ve^{-\theta^\top|t_n-t_1|}\\
e^{-\theta|t_2-t_1|}V & V & \dots & Ve^{-\theta^\top|t_{n-1}-t_2|} & Ve^{-\theta^\top|t_n-t_2|}\\
\vdots & \vdots & \ddots & \vdots & \vdots\\
e^{-\theta|t_{n-1}-t_1|}V & e^{-\theta|t_{n-1}-t_2|}V & \dots & V & Ve^{-\theta^\top|t_{n-1}-t_n|}\\
e^{-\theta|t_n-t_1|}V & e^{-\theta|t_n-t_2|}V & \dots & e^{-\theta|t_n-t_{n-1}|}V & V
\end{bmatrix}$$

By the definition of the OU process, we know that the precision matrix, $\Omega=\Psi^{-1}$, is block tri-diagonal. We start by solving for two blocks, Ω_{11} and Ω_{12}. We assume that $\Omega_{11}=A^{-1}$ and $\Omega_{12}=A^{-1}B$, based on the form of the precision matrix in the case of the univariate OU process. Based on patterns seen when multiplying the AR(1) precision and covariance matrices, we assume that, for the OU process, the first row of blocks in the precision matrix, $[\Omega_{11},\Omega_{12},0,\dots,0]$ times the second column of blocks in the covariance matrix, $[Ve^{-\theta^\top(t_2-t_1)},V,\dots]^\top$, is equal to 0. So,

$$\begin{aligned}
0 &= \Omega_{11}Ve^{-\theta^\top(t_2-t_1)}+\Omega_{12}V\\
\implies 0 &= A^{-1}Ve^{-\theta^\top(t_2-t_1)}+A^{-1}BV\\
\implies 0 &= Ve^{-\theta^\top(t_2-t_1)}+BV\\
\implies BV &= -Ve^{-\theta^\top(t_2-t_1)}\\
\implies B &= -Ve^{-\theta^\top(t_2-t_1)}V^{-1}
\end{aligned}$$

By similar logic, the first row of blocks in the precision matrix times the first column of

blocks in the covariance matrix is equal to the identity matrix. So,

$$
\begin{aligned}
I &= \Omega_{11} V + \Omega_{12} e^{-\theta(t_2-t_1)} V \\
\implies I &= A^{-1} V + A^{-1} B e^{-\theta(t_2-t_1)} V \\
\implies A &= V + B e^{-\theta(t_2-t_1)} V
\end{aligned}
$$

We know that $B = -V e^{-\theta^\top(t_2-t_1)} V^{-1}$ so

$$
\begin{aligned}
A &= V - V e^{-\theta^\top(t_2-t_1)} V^{-1} e^{-\theta(t_2-t_1)} V \\
\implies A^{-1} &= [V - V e^{-\theta^\top(t_2-t_1)} V^{-1} e^{-\theta(t_2-t_1)} V]^{-1}
\end{aligned}
$$

Now we have

$$
\begin{aligned}
\Omega_{11} &= \left[V - V e^{-\theta^\top(t_2-t_1)} V^{-1} e^{-\theta(t_2-t_1)} V\right]^{-1} \\
\Omega_{12} &= -\left[V - V e^{-\theta^\top(t_2-t_1)} V^{-1} e^{-\theta(t_2-t_1)} V\right]^{-1} V e^{-\theta^\top(t_2-t_1)} V^{-1}
\end{aligned}
$$

Continuing with this logic, we can check the first row of blocks in Ω against all other columns of Ψ and see that

$$
\begin{aligned}
0 &= \Omega_{11} V e^{-\theta^\top(t_k-t_1)} + \Omega_{12} V e^{-\theta^\top(t_k-t_2)} \\
&= A^{-1} V e^{-\theta^\top(t_k-t_1)} + A^{-1} B V e^{-\theta^\top(t_k-t_2)} \\
&= V e^{-\theta^\top(t_k-t_1)} + B V e^{-\theta^\top(t_k-t_2)} \\
&= V e^{-\theta^\top(t_k-t_1)} - V e^{-\theta^\top(t_2-t_1)} V^{-1} V e^{-\theta^\top(t_k-t_2)} \\
&= V e^{-\theta^\top(t_k-t_1)} - V e^{-\theta^\top(t_2-t_1)} e^{-\theta^\top(t_k-t_2)} \\
&= V e^{-\theta^\top(t_k-t_1)} - V e^{-\theta^\top(t_k-t_1)} \\
&= 0
\end{aligned}
$$

Now we move to the second row of blocks in Ω. Because $\Omega = \Omega^\top$, we also know that $\Omega_{21} = \Omega_{12}^\top$. This symmetry means that we only need to derive Ω_{22} and Ω_{23}. Based on previous results, we have

$$
\Omega_{23} = -\left[V - V e^{-\theta^\top(t_3-t_2)} V^{-1} e^{-\theta(t_3-t_2)} V\right]^{-1} V e^{-\theta^\top(t_3-t_2)} V^{-1}
$$

Then we find the form of Ω_{22} by once again using the same logic to say that the second

row of blocks in Ω times the second column of blocks in Ψ will be equal to an identity matrix:

$$
\begin{aligned}
I &= \Omega_{21} V e^{-\theta^\top (t_2 - t_1)} + \Omega_{22} V + \Omega_{23} e^{-\theta(t_3 - t_2)} V \\
\Rightarrow V^{-1} &= \Omega_{21} V e^{-\theta^\top (t_2 - t_1)} V^{-1} + \Omega_{22} + \Omega_{23} e^{-\theta(t_3 - t_2)} \\
\Rightarrow \Omega_{22} &= V^{-1} + V^{-1} e^{-\theta(t_2 - t_1)} V \left[V - V e^{-\theta^\top (t_2 - t_1)} V^{-1} e^{-\theta(t_2 - t_1)} V \right]^{-1^\top} V e^{-\theta^\top (t_2 - t_1)} V^{-1} \\
&\quad + \left[V - V e^{-\theta^\top (t_3 - t_2)} V^{-1} e^{-\theta(t_3 - t_2)} V \right]^{-1} V e^{-\theta^\top (t_3 - t_2)} V^{-1} e^{-\theta(t_3 - t_2)}
\end{aligned}
$$

The final terms are then given by:

$$
\begin{aligned}
I &= \Omega_{n,n-1} V e^{-\theta^\top (t_n - t_{n-1})} + \Omega_{nn} V \\
\Rightarrow I &= -V^{-1} e^{-\theta(t_n - t_{n-1})} V \left[V - V e^{\theta^\top (t_n - t_{n-1})} V^{-1} e^{-\theta(t_n - t_{n-1})} V \right]^{-1} V e^{-\theta^\top (t_n - t_{n-1})} + \Omega_{nn} V \\
\Rightarrow \Omega_{nn} &= V^{-1} + V^{-1} e^{-\theta(t_n - t_{n-1})} V \left[V - V e^{-\theta^\top (t_n - t_{n-1})} V^{-1} e^{-\theta(t_n - t_{n-1})} V \right]^{-1} V e^{-\theta^\top (t_n - t_{n-1})} V^{-1}
\end{aligned}
$$

Thus, the precision matrix Ω is block tri-diagonal with the following entries (indexed by j) for $1 < j < n$:

$$
\begin{aligned}
V &:= vec^{-1}\big\{(\theta \oplus \theta)^{-1} vec\{\sigma\sigma^\top\}\big\} \\
\Omega_{11} &= \big[V - V e^{-\theta^\top (t_2 - t_1)} V^{-1} e^{-\theta(t_2 - t_1)} V\big]^{-1} \\
\Omega_{j,j+1} &= \Omega_{j+1,j}^\top = -\big[V - V e^{-\theta^\top (t_{j+1} - t_j)} V^{-1} e^{-\theta(t_{j+1} - t_j)} V\big]^{-1} V e^{-\theta^\top (t_{j+1} - t_j)} V^{-1} \\
\Omega_{jj} &= V^{-1} + V^{-1} e^{-\theta(t_j - t_{j-1})} V \big[V - V e^{-\theta^\top (t_j - t_{j-1})} V^{-1} e^{-\theta(t_j - t_{j-1})} V\big]^{-1} V e^{-\theta^\top (t_j - t_{j-1})} V^{-1} \\
&\qquad + \big[V - V e^{-\theta^\top (t_{j+1} - t_j)} V^{-1} e^{-\theta(t_{j+1} - t_j)} V\big]^{-1} V e^{-\theta^\top (t_{j+1} - t_j)} V^{-1} e^{-\theta(t_{j+1} - t_j)} \\
\Omega_{nn} &= V^{-1} + V^{-1} e^{-\theta(t_n - t_{n-1})} V \big[V - V e^{-\theta^\top (t_n - t_{n-1})} V^{-1} e^{-\theta(t_n - t_{n-1})} V\big]^{-1} V e^{-\theta^\top (t_n - t_{n-1})} V^{-1}
\end{aligned}
$$

A.4 Identifiability constraint: re-scaling the OU process

Let (θ^*, σ^*) be a pair of OU process parameters satisfying the identifiability constraint that the stationary variance of the OU process is equal to 1; that is, $diag\{\Psi(\theta^*, \sigma^*)\} = 1$, where Ψ is the covariance matrix of the OU process. We show that we can always find a pair of (θ^*, σ^*) that defines a valid mean-reverting OU process with stationary variance of 1 that has the same correlation structure as the original unconstrained OU process defined by (θ, σ). As an example, consider the stochastic differential equation definition of the bivariate OU process. For an arbitrary mean-reverting OU process, $\eta(t)$,

$$
d \begin{bmatrix} \eta_1(t) \\ \eta_2(t) \end{bmatrix} = - \begin{bmatrix} \theta_{11} & \theta_{12} \\ \theta_{21} & \theta_{22} \end{bmatrix} \begin{bmatrix} \eta_1(t) \\ \eta_2(t) \end{bmatrix} dt + \begin{bmatrix} \sigma_{11} & 0 \\ 0 & \sigma_{22} \end{bmatrix} d \begin{bmatrix} W_1(t) \\ W_2(t) \end{bmatrix}
$$

We could equivalently define this OU process $\eta(t)$ as

$$d\begin{bmatrix}\eta_1(t)\\ \eta_2(t)\end{bmatrix} = -\begin{bmatrix}\theta_{11} & \theta_{12}\\ \theta_{21} & \theta_{22}\end{bmatrix}\begin{bmatrix}c_1 & 0\\ 0 & c_2\end{bmatrix}\begin{bmatrix}1/c_1 & 0\\ 0 & 1/c_2\end{bmatrix}\begin{bmatrix}\eta_1(t)\\ \eta_2(t)\end{bmatrix}dt + \begin{bmatrix}\sigma_{11} & 0\\ 0 & \sigma_{22}\end{bmatrix}d\begin{bmatrix}W_1(t)\\ W_2(t)\end{bmatrix}$$
$$= -\begin{bmatrix}c_1\theta_{11} & c_2\theta_{12}\\ c_1\theta_{21} & c_2\theta_{22}\end{bmatrix}\begin{bmatrix}\frac{1}{c_1}\eta_1(t)\\ \frac{1}{c_2}\eta_2(t)\end{bmatrix}dt + \begin{bmatrix}\sigma_{11} & 0\\ 0 & \sigma_{22}\end{bmatrix}d\begin{bmatrix}W_1(t)\\ W_2(t)\end{bmatrix}$$

Let $\eta^*(t)$ be a scaled version of η where

$$\begin{bmatrix}\eta_1^*(t)\\ \eta_2^*(t)\end{bmatrix} = \begin{bmatrix}\frac{1}{c_1}\eta_1(t)\\ \frac{1}{c_2}\eta_2(t)\end{bmatrix}$$

and

$$\begin{bmatrix}\theta_{11}^* & \theta_{12}^*\\ \theta_{21}^* & \theta_{22}^*\end{bmatrix} = \begin{bmatrix}c_1\theta_{11} & c_2\theta_{12}\\ c_1\theta_{21} & c_2\theta_{22}\end{bmatrix}$$

and assume that $\eta^*(t)$ has a stationary variance equal to 1. Then,

$$d\eta^*(t) = -\begin{bmatrix}\theta_{11}^* & \theta_{21}^*\\ \theta_{12}^* & \theta_{22}^*\end{bmatrix}\begin{bmatrix}\eta_1^*(t)\\ \eta_2^*(t)\end{bmatrix}dt + \begin{bmatrix}\sigma_{11}^* & 0\\ 0 & \sigma_{22}^*\end{bmatrix}d\begin{bmatrix}W_1(t)\\ W_2(t)\end{bmatrix}$$
$$= -\begin{bmatrix}\theta_{11}^* & \theta_{21}^*\\ \theta_{12}^* & \theta_{22}^*\end{bmatrix}\begin{bmatrix}c_1 & 0\\ 0 & c_2\end{bmatrix}\begin{bmatrix}\eta_1(t)\\ \eta_2(t)\end{bmatrix}dt + \begin{bmatrix}\sigma_{11}^* & 0\\ 0 & \sigma_{22}^*\end{bmatrix}d\begin{bmatrix}W_1(t)\\ W_2(t)\end{bmatrix}$$
$$= -\begin{bmatrix}c_1\theta_{11}^* & c_2\theta_{21}^*\\ c_1\theta_{12}^* & c_2\theta_{22}^*\end{bmatrix}\begin{bmatrix}\eta_1(t)\\ \eta_2(t)\end{bmatrix}dt + \begin{bmatrix}\sigma_{11}^* & 0\\ 0 & \sigma_{22}^*\end{bmatrix}d\begin{bmatrix}W_1(t)\\ W_2(t)\end{bmatrix}$$

Looking back at the original OU process $\eta(t)$,

$$d\eta(t) = d\begin{bmatrix}\frac{1}{c_1} & 0\\ 0 & \frac{1}{c_2}\end{bmatrix}\eta^*(t)$$
$$= -\begin{bmatrix}\frac{1}{c_1} & 0\\ 0 & \frac{1}{c_2}\end{bmatrix}\begin{bmatrix}c_1\theta_{11}^* & c_2\theta_{12}^*\\ c_1\theta_{21}^* & c_2\theta_{22}^*\end{bmatrix}\eta(t)dt + \begin{bmatrix}\frac{1}{c_1}\sigma_{11}^* & 0\\ 0 & \frac{1}{c_2}\sigma_{22}^*\end{bmatrix}dW(t)$$
$$= -\begin{bmatrix}\frac{c_1}{c_1}\theta_{11}^* & \frac{c_2}{c_1}\theta_{12}^*\\ \frac{c_1}{c_2}\theta_{21}^* & \frac{c_2}{c_2}\theta_{22}^*\end{bmatrix}\eta(t)dt + \begin{bmatrix}\frac{1}{c_1}\sigma_{11}^* & 0\\ 0 & \frac{1}{c_2}\sigma_{22}^*\end{bmatrix}dW(t)$$

Finally, we see that the parameters for $\eta(t)$ can easily be re-scaled to satisfy our identifiability assumption:

$$\begin{bmatrix}\theta_{11} & \frac{c_1}{c_2}\theta_{12}\\ \frac{c_2}{c_1}\theta_{21} & \theta_{22}\end{bmatrix} = \begin{bmatrix}\theta_{11}^* & \theta_{12}^*\\ \theta_{21}^* & \theta_{22}^*\end{bmatrix}$$

and

$$\begin{bmatrix}c_1\sigma_{11} & 0\\ 0 & c_2\sigma_{22}\end{bmatrix} = \begin{bmatrix}\sigma_{11}^* & 0\\ 0 & \sigma_{22}^*\end{bmatrix}$$

Thus, we have shown that for a mean-reverting bivariate OU process defined by θ and σ with covariance matrix $\Psi(\theta, \sigma)$ and correlation matrix $\Psi^*(\theta, \sigma)$, we can re-scale this OU process to have stationary variance equal to 1 by scaling θ_{12}, θ_{21} and σ_{11}, σ_{22} by a pair of

positive scalar constants, (c_1, c_2). This proof can easily be extended to higher dimensional OU processes.

A.5 Derivation of the analytic gradients for the measurement submodel

We have previously defined the log-likelihood for a single subject i as

$$\ell_i = -\frac{1}{2} log|\Sigma_i^*| + Y_i^\top \Sigma_i^{*-1} Y_i \tag{7}$$

where we ignore the constant terms and

$$\Sigma_i^* = (I_{n_i} \otimes \Lambda)\Psi_i(I_{n_i} \otimes \Lambda)^\top + J_{n_i} \otimes \Sigma_u + I_{n_i} \otimes \Sigma_\epsilon \tag{8}$$

Gradient w.r.t. the loadings: We first take the derivative of ℓ_i with respect to the elements of the loadings matrix Λ, λ_k, $k = 1, ..., p \times K$. The first element of the loadings matrix is parameterized on the log scale in order to restrict this element to positive values for identifiability purposes and so the gradient of this element looks slightly different. For $k > 1$, we have

$$\frac{\partial \ell_i}{\partial \lambda_k} = -\frac{1}{2}\left[tr\left\{ \Sigma_i^{*-1} \frac{\partial \Sigma_i^*}{\partial \lambda_k} \right\} - Y_i^\top \Sigma_i^{*-1} \frac{\partial \Sigma_i^*}{\partial \lambda_k} \Sigma_i^{*-1} Y_i \right] \tag{9}$$

where

$$\frac{\partial \Sigma_i^*}{\partial \lambda_k} = (I_{n_i} \otimes \Lambda)\Psi_i(I_{n_i} \otimes J^{k^\top}) + (I_{n_i} \otimes J^k)\Psi_i(I_{n_i} \otimes \Lambda^\top) \tag{10}$$

We use J^k as an indicator matrix that has the same dimension as Λ but contains all zeros except for a single 1 indicating the location of element λ_k. For $k = 1$, we apply the chain rule and have

$$\frac{\partial \ell_i}{\partial log(\lambda_k)} = \frac{\partial \ell_i}{\partial \lambda_k}\left[\frac{\partial log(\lambda_k)}{\partial \lambda_k} \right]^{-1} = \frac{\partial \ell_i}{\partial \lambda_k}\lambda_k \tag{11}$$

Gradient w.r.t. the random effects: Next, we take the gradient of ℓ_i with respect to the elements of R_u where R_u comes from the Cholesky decomposition of the random effects covariance matrix, $\Sigma_u = R_u^\top R_u$. For $p, q = 1, ..., K$ and $p \neq q$,

$$\frac{\partial \Sigma_i^*}{\partial r_{pq}} = J_{n_i} \otimes (J^{k^\top} R_u + R_u^\top J^k) \tag{12}$$

$$\frac{\partial \ell_i}{\partial r_{pq}} = -\frac{1}{2}\left[tr\left\{ \Sigma_i^{*-1} \frac{\partial \Sigma_i^*}{\partial r_{pq}} \right\} + Y_i^\top \Sigma_i^{*-1} \frac{\partial \Sigma_i^*}{\partial r_{pq}} \Sigma_i^{*-1} Y_i \right] \tag{13}$$

where again J^k is an indicator matrix of the same dimensions as Σ_u.

For $p, q = 1, ..., K$ and $p = q$,

$$\frac{\partial \ell_i}{\partial log(r_{u_{pp}})} = \frac{\partial \ell_i}{\partial r_{u_{pp}}}\left[\frac{\partial log(r_{u_{pp}})}{\partial r_{u_{pp}}}\right]^{-1} = \frac{\partial \ell_i}{\partial r_{u_{pp}}} r_{u_{pp}} \tag{14}$$

Note that if we assume only random intercepts (i.e., a diagonal covariance matrix) then we can avoid the Cholesky decomposition by estimating σ_u on the log scale. In this case, the gradient simplifies to the form given below for the measurement error.

Gradient w.r.t. the measurement error: Finally, we take the gradient of ℓ_i with respect to the elements of the measurement error covariance matrix, Σ_ϵ. For $k = 1, ..., K$, we have

$$\frac{\partial \Sigma_i^*}{\partial \sigma_{\epsilon_k}} = I_{n_i} \otimes 2\sigma_{\epsilon_k} J^k \tag{15}$$

$$\frac{\partial \ell_i}{\partial \sigma_{\epsilon_k}} = -\frac{1}{2}\left[tr\left\{\Sigma_i^{*-1}\frac{\partial \Sigma_i^*}{\partial \sigma_{\epsilon_k}}\right\} - Y_i^\top \Sigma_i^{*-1}\frac{\partial \Sigma_i^*}{\partial \sigma_{\epsilon_k}}\Sigma_i^{*-1} Y_i\right] \tag{16}$$

$$\frac{\partial \ell_i}{\partial log(\sigma_{\epsilon_k})} = \frac{\partial \ell_i}{\partial \sigma_{\epsilon_k}}\left[\frac{log(\sigma_{\epsilon_k})}{\partial \sigma_{\epsilon_k}}\right]^{-1} = \frac{\partial \ell_i}{\partial \sigma_{\epsilon_k}}\sigma_{\epsilon_k} \tag{17}$$

where J^k is an indicator matrix of the same dimensions as Σ_ϵ.

A.6 Parameterization of the log-likelihood for standard error estimation

To make our OUF model identifiable, we impose a constraint on the scale of the OU process by forcing the stationary variance equal to 1 via a set of p positive scalar constants. These constants are functions of OU parameters θ and σ.

When the log-likelihood is allowed to vary as a function all parameters, rather than just a single block of parameters as in our block coordinate descent algorithm, our model is no longer identifiable. To estimate standard errors, we take advantage of the fact that under the identifiability constraint, σ can be written as a function of θ, as shown here:

Recall that the stationary variance of the OU process is $V := vec^{-1}\Big\{(\theta \oplus \theta)^{-1} vec\{\sigma\sigma^\top\}\Big\}$. Assuming a bivariate OU process, under the identifiability constraint, V takes the form $\begin{bmatrix} 1 & \rho \\ \rho & 1 \end{bmatrix}$ where the off-diagonal element ρ is the correlation. Then,

$$\begin{bmatrix} 1 & \rho \\ \rho & 1 \end{bmatrix} = vec^{-1}\Big\{(\theta \oplus \theta)^{-1} vec\{\sigma\sigma^{-1}\}\Big\} \Longrightarrow \begin{bmatrix} 1 \\ \rho \\ \rho \\ 1 \end{bmatrix} = (\theta \oplus \theta)^{-1}\begin{bmatrix} \sigma_{11}^2 \\ 0 \\ 0 \\ \sigma_{22}^2 \end{bmatrix}.$$

Letting

$$(\theta \oplus \theta)^{-1} = \begin{bmatrix} x_{11} & x_{12} & x_{13} & x_{14} \\ x_{21} & x_{22} & x_{23} & x_{24} \\ x_{31} & x_{32} & x_{33} & x_{34} \\ x_{41} & x_{42} & x_{43} & x_{44} \end{bmatrix},$$

where each element x_{ij} is some function of the elements of θ, we can solve for σ in the (θ, σ) pair that satisfies the identifiability constraint via

$$1 = x_{11}\sigma_{11}^2 + x_{14}\sigma_{22}^2$$
$$1 = x_{41}\sigma_{11}^2 + x_{44}\sigma_{22}^2$$

By constraining σ to be a function of θ, we take an alternative approach to identification and no longer require use of the scaling constants here.

A.7 Choice of true OU process in simulation study

In the simulation study described in the main text (Section 4.1-4.2), we generate data in three different settings in which the true OU process has varying degrees of auto-correlation. These parameters are used in both the ILD and non-ILD simulations. We present the true OU process parameters here:

Setting 1:

$$\theta = \begin{bmatrix} 1 & 0.6 \\ 4 & 5 \end{bmatrix} \text{ and } \sigma = \begin{bmatrix} 1 & 0 \\ 0 & 2 \end{bmatrix}$$

Setting 2:

$$\theta = \begin{bmatrix} 1.0 & 0.4 \\ 1.8 & 3.0 \end{bmatrix} \text{ and } \sigma = \begin{bmatrix} 1.25 & 0 \\ 0 & 2.00 \end{bmatrix}$$

Setting 3:

$$\theta = \begin{bmatrix} 1 & 0.5 \\ 2 & 5 \end{bmatrix} \text{ and } \sigma = \begin{bmatrix} 2 & 0 \\ 0 & 3 \end{bmatrix}$$

In the simulation study assessing use of AIC and BIC to select the correct number of latent factors in a model (described in the main text in Section 4.3-4.4), the true parameters were set to the values listed below. The true values used for Σ_u and Σ_ϵ were the same as in the original simulation study (see Section 4.1 in the main text).

One factor model:

$$\Lambda = \begin{bmatrix} 1.2 \\ 1.8 \\ -0.4 \\ 2 \end{bmatrix}, \quad \theta = 0.8, \quad \sigma = 1$$

Two factor model with low signal:

$$\Lambda = \begin{bmatrix} 1.2 & 0 \\ 1.8 & 0 \\ 0 & -0.4 \\ 0 & 2 \end{bmatrix}, \quad \theta = \begin{bmatrix} 2 & 0.5 \\ 0.4 & 4 \end{bmatrix}, \quad \sigma = \begin{bmatrix} 2 & 0 \\ 0 & 1 \end{bmatrix}$$

Two factor model with high signal:

$$\Lambda = \begin{bmatrix} 1.2 & 0 \\ 1.8 & 0 \\ 0 & -0.4 \\ 0 & 2 \end{bmatrix}, \quad \theta = \begin{bmatrix} 1 & 1.5 \\ 2 & 5 \end{bmatrix}, \quad \sigma = \begin{bmatrix} 2 & 0 \\ 0 & 3 \end{bmatrix}$$

Three factor model with low signal:

$$\Lambda = \begin{bmatrix} 1.2 & 0 & 0 \\ 1.8 & 0 & 0 \\ 0 & -0.4 & 0 \\ 0 & 0 & 2 \end{bmatrix}, \quad \theta = \begin{bmatrix} 2 & 0.2 & 0.4 \\ 0.8 & 1.1 & 0.5 \\ 0.7 & 0.5 & 1.2 \end{bmatrix}, \quad \sigma = \begin{bmatrix} 1.2 & 0 & 0 \\ 0 & 0.8 & 0 \\ 0 & 0 & 0.4 \end{bmatrix}$$

Three factor model with high signal:

$$\Lambda = \begin{bmatrix} 1.2 & 0 & 0 \\ 1.8 & 0 & 0 \\ 0 & -0.4 & 0 \\ 0 & 0 & 2 \end{bmatrix}, \quad \theta = \begin{bmatrix} 1 & 0.4 & 0.6 \\ 1.8 & 3 & 0.9 \\ 0.9 & 1 & 1.2 \end{bmatrix}, \quad \sigma = \begin{bmatrix} 1.2 & 0 & 0 \\ 0 & 0.8 & 0 \\ 0 & 0 & 0.4 \end{bmatrix}$$

When fitting the models in our simulation studies, we assume that the loadings matrices take the same structure as above (i.e., the locations of the structural zeros are specified as above for all fitted one-, two-, and three-factor models). In our model selection simulation study, this means that if the fitted model and data-generating model have the same number of factors, the location of the structural zeros in the fitted model will match the true model. If the fitted model and the data-generating model differ in the number of latent factors, then the loadings matrix of the fitted model will not have structural zeros in the correct locations.

A.8 Results from simulation study with ILD

Simulation study: bias and variance In Supplementary Tables 1, 2, and 3, we summarize the results of our simulation study by reporting average relative bias (reported as (estimate - truth) / truth x 100), root mean squared error (RMSE), the empirical standard deviation of the point estimates across simulation replicates, the average standard error, and the coverage rate of 95% confidence intervals.

The estimation algorithm failed to converge due to numerical issues when applied to a few of the simulated datasets generated in the simulation study described in Section 4.1-4.2. The failures were caused by a singular V matrix at the start of the first block update of the structural submodel parameters. Slightly altering the values at which the OU process parameters were initialized resolved this issue. Point estimates were ultimately calculate for all 1000 simulated datasets in each setting. In Setting 3, an invalid variance for the measurement submodel parameter $\sigma^2_{\epsilon_4}$ was estimated from one dataset. In this instance, the variance estimated for this parameter was negative. We attribute this issue to the numerical approximation used to calculate the Hessian when applied to these this dataset of size $N = 200$. We anticipate that a larger dataset would improve the approximation of the numerical Hessian but chose to simulate a dataset of this size in order to assess model performance in a realistic setting similar to that encountered in the motivating data application. In practical application, if a negative variance were to be estimated, it could be rounded to 0. In the results presented in the main text, we ignore the variance estimate for this one $\sigma^2_{\epsilon_4}$.

Simulation study: model selection In our simulation studies, we aimed to assess simulated datasets with sample sizes similar to that of our motivating dataset. For datasets of fixed size ($N = 200$ subjects), we found that convergence speeds decrease and estimation becomes more difficult as the number of factors in the model increases. We found that point estimates of the diagonal elements of θ_{OU} hit the lower bound of 1×10^{-4} less than 1% of the time. To improve convergence, we slightly altered the set of default parameter values considered during the initialization steps of the block-wise estimation algorithm for a subset of datasets. However, when assessing AIC and BIC as model selection criteria (see Section 4.3-4.4), we very occasionally encountered numerical issues and so failed to calculate parameter estimates for a subset of models applied to the simulated datasets. The results reported in the main paper correspond to a comparison of AIC and BIC across datasets for which the algorithm used to fit all three models (the one-factor, two-factor, and three-factor models) either converged or reached the maximum number of iterations prior to convergence. We assessed whether or not including results in which the maximum number of iterations was reached prior to convergence impacted our model selection results and found no substantial changes. Supplementary Table 4 shows the equivalent version of Table 1 presented in the main text if only results from datasets that had converged were shown.

In Supplementary Table 5, we summarize the number (out of 100) of datasets (in each setting) for which the algorithm converged (using $\delta = 1 \times 10^{-6}$) or reached the maximum number of iterations prior to convergence. When this total number does not add up to 100, the remaining datasets correspond to situations in which the algorithm failed due to numerical issues (e.g., current OU parameter estimates corresponded to a singular stationary

Param.	Truth	Relative bias (x 100)	RMSE	Empirical SD	SE	Coverage rate (%)
λ_1	1.16	-0.34	0.03	0.032	0.031	94.6
λ_2	1.74	-0.37	0.04	0.044	0.042	94.2
λ_3	-0.39	-0.29	0.02	0.017	0.017	94.9
λ_4	1.97	-0.30	0.05	0.053	0.051	93.8
$\sigma^2_{u_1}$	1.10	-0.41	0.13	0.127	0.130	94.2
$\sigma^2_{u_2}$	1.30	-0.59	0.17	0.175	0.173	94.2
$\sigma^2_{u_3}$	1.40	-1.27	0.14	0.143	0.143	93.4
$\sigma^2_{u_4}$	0.90	-0.74	0.14	0.143	0.142	94.1
$\sigma^2_{e_1}$	0.60	-0.57	0.02	0.022	0.022	95.0
$\sigma^2_{e_2}$	0.50	-0.40	0.04	0.037	0.035	93.5
$\sigma^2_{e_3}$	0.40	-0.21	0.01	0.012	0.012	94.4
$\sigma^2_{e_4}$	0.70	-1.05	0.12	0.122	0.123	94.8
$\theta_{OU_{11}}$	1.00	-0.50	0.19	0.187	0.182	95.1
$\theta_{OU_{21}}$	3.91	2.14	0.60	0.596	0.612	95.9
$\theta_{OU_{12}}$	0.61	-1.25	0.21	0.212	0.208	95.3
$\theta_{OU_{22}}$	5.00	2.10	0.75	0.741	0.765	96.4
$\sigma_{OU_{11}}$	1.04	-0.07	0.04			
$\sigma_{OU_{22}}$	2.03	0.66	0.16			

Supplementary Table 1: ILD setting 1. Across the 1000 simulation replicates, we report the average relative bias ((estimate - truth) / truth), root mean squared error (RMSE), empirical standard deviation (SD) of the point estimates, the average estimated standard error (SE), and the coverage rate of the 95% confidence intervals. Note that average relative bias is scaled up by a factor of 100 and that coverage rate is reported as a percent.

Param.	Truth	Relative bias (x 100)	RMSE	Empirical SD	SE	Coverage rate (%)
λ_1	1.20	-0.35	0.03	0.029	0.029	94.4
λ_2	1.80	-0.36	0.04	0.040	0.039	94.2
λ_3	-0.40	-0.21	0.02	0.017	0.017	95.4
λ_4	2.00	-0.35	0.06	0.055	0.054	93.7
$\sigma^2_{u_1}$	1.10	-0.42	0.13	0.128	0.130	94.3
$\sigma^2_{u_2}$	1.30	-0.59	0.18	0.175	0.174	94.6
$\sigma^2_{u_3}$	1.40	-1.28	0.14	0.143	0.143	93.4
$\sigma^2_{u_4}$	0.90	-0.78	0.14	0.137	0.137	94.2
$\sigma^2_{e_1}$	0.60	-0.58	0.03	0.026	0.026	95.7
$\sigma^2_{e_2}$	0.50	-0.44	0.05	0.049	0.046	93.8
$\sigma^2_{e_3}$	0.40	-0.27	0.01	0.012	0.013	94.9
$\sigma^2_{e_4}$	0.70	-0.63	0.16	0.160	0.162	95.3
$\theta_{OU_{11}}$	1.00	0.14	0.10	0.103	0.100	93.8
$\theta_{OU_{21}}$	1.80	1.15	0.22	0.217	0.215	95.0
$\theta_{OU_{12}}$	0.40	-0.44	0.12	0.120	0.117	94.6
$\theta_{OU_{22}}$	3.00	1.00	0.33	0.328	0.328	95.3
$\sigma_{OU_{11}}$	1.25	0.04	0.05			
$\sigma_{OU_{22}}$	2.00	0.24	0.12			

Supplementary Table 2: ILD setting 2. Across the 1000 simulation replicates, we report the average relative bias ((estimate - truth) / truth), root mean squared error (RMSE), empirical standard deviation (SD) of the point estimates, the average estimated standard error (SE), and the coverage rate of the 95% confidence intervals. Note that average relative bias is scaled up by a factor of 100 and that coverage rate is reported as a percent.

Param.	Truth	Relative bias (x 100)	RMSE	Empirical SD	SE	Coverage rate (%)
λ_1	1.88	-0.35	0.04	0.038	0.038	94.6
λ_2	2.83	-0.37	0.06	0.054	0.054	94.7
λ_3	-0.45	-0.30	0.02	0.018	0.018	94.8
λ_4	2.25	-0.29	0.06	0.063	0.062	93.8
$\sigma^2_{u_1}$	1.10	-0.39	0.14	0.140	0.142	94.0
$\sigma^2_{u_2}$	1.30	-0.59	0.21	0.215	0.213	94.6
$\sigma^2_{u_3}$	1.40	-1.28	0.14	0.143	0.143	93.4
$\sigma^2_{u_4}$	0.90	-0.73	0.14	0.135	0.134	94.0
$\sigma^2_{e_1}$	0.60	-0.60	0.03	0.034	0.033	94.9
$\sigma^2_{e_2}$	0.50	-0.37	0.07	0.071	0.067	94.1
$\sigma^2_{e_3}$	0.40	-0.23	0.01	0.014	0.014	95.3
$\sigma^2_{e_4}$	0.70	-1.70	0.22	0.224	0.224	94.9
$\theta_{OU_{11}}$	1.00	-0.05	0.11	0.110	0.107	94.2
$\theta_{OU_{21}}$	2.78	1.79	0.40	0.397	0.397	94.5
$\theta_{OU_{12}}$	0.36	-1.27	0.16	0.157	0.154	95.2
$\theta_{OU_{22}}$	5.00	1.72	0.68	0.673	0.675	95.2
$\sigma_{OU_{11}}$	1.27	0.03	0.04			
$\sigma_{OU_{22}}$	2.66	0.57	0.19			

Supplementary Table 3: ILD setting 3. Across the 1000 simulation replicates, we report the average relative bias ((estimate - truth) / truth), root mean squared error (RMSE), empirical standard deviation (SD) of the point estimates, the average estimated standard error (SE), and the coverage rate of the 95% confidence intervals. Note that average relative bias is scaled up by a factor of 100 and that coverage rate is reported as a percent.

True Model		# Factors in Fitted Model with Best AIC			# Factors in Fitted Model with Best BIC		
# Factors	Signal	1	2	3	1	2	3
1	-	**99**	0	1	**100**	0	0
2	Low	0	**93**	7	4	**96**	0
2	High	0	**100**	0	0	**100**	0
3	Low	0	0	**100**	0	8	**92**
3	High	0	0	**100**	0	0	**100**

Supplementary Table 4: For datasets generated under each true model, we summarize the percent of times that the model-selection metric chose the fitted model with the indicated number of factors. The settings in which the fitted model has the same number of factors as the true data-generating model are emphasized with bold orange text. These results are presented for datasets on which the algorithm converged prior to reaching the maximum number of iterations (200) for all three models.

		# Factors in Fitted Model					
True Model		1		2		3	
# Factors	Signal	convergence	iteration limit	convergence	iteration limit	convergence	iteration limit
1	-	100	0	100	0	79	20
2	Low	100	0	100	0	96	4
2	High	100	0	100	0	98	2
3	Low	100	0	99	1	100	0
3	High	99	0	100	0	99	1

Supplementary Table 5: For datasets generated under each true model, we summarize the number of datasets (out of 100) on which the algorithm converged or reached the maximum number of block-wise iterations prior to convergence (when $\delta = 1 \times 10^{-6}$). For totals that do not sum to 100, the remaining cases correspond to instances in which the algorithm failed due to numerical issues prior to converging or reaching the maximum number of block-wise iterations (200).

		# Factors in Fitted Model					
True Model		1		2		3	
# Factors	Signal	convergence	iteration limit	convergence	iteration limit	convergence	iteration limit
1	-	100	0	100	0	94	5
2	Low	100	0	100	0	100	0
2	High	100	0	100	0	100	0
3	Low	100	0	99	1	100	0
3	High	99	0	100	0	100	0

Supplementary Table 6: For datasets generated under each true model, we summarize the number of datasets (out of 100) on which the algorithm converged or reached the maximum number of block-wise iterations prior to convergence (when $\delta \leq 1 \times 10^{-3}$). For totals that do not sum to 100, the remaining cases correspond to instances in which the algorithm failed due to numerical issues prior to converging or reaching the maximum number of block-wise iterations (200).

covariance matrix).

After loosening the convergence criteria across the block-wise iterations, we did not find substantially different results when evaluating AIC and BIC as model selection criteria when compared to results under the original convergence criteria. For example, if we categorized convergence using $\delta \leq 1 \times 10^{-3}$, rather than only the original $\delta = 1 \times 10^{-6}$, the algorithm would have converged when fitting almost every model to almost every dataset (see Supplementary Table 6) but the model selection results would not have changed (see Supplementary Table 7).

We expect that increasing the size of the simulated dataset would increase the rate at which we successfully fit models with more factors.

A.9 Results from simulation study with non-ILD

We present results from the simulation study with non-ILD in Supplementary Figures 1 and 2. As with the ILD simulation study, the estimation algorithm failed to converge due to numerical issues when applied to a few of the simulated datasets. As before, slightly

True Model		# Factors in Fitted Model with Best AIC			# Factors in Fitted Model with Best BIC		
**# Factors**	**Signal**	**1**	**2**	**3**	**1**	**2**	**3**
1	-	**99**	0	1	**100**	0	0
2	Low	0	**93**	7	4	**96**	0
2	High	0	**100**	0	0	**100**	0
3	Low	0	0	**100**	0	8	**92**
3	High	0	0	**100**	0	0	**100**

Supplementary Table 7: For datasets generated under each true model, we summarize the percent of times that the model-selection metric chose the fitted model with the indicated number of factors. The settings in which the fitted model has the same number of factors as the true data-generating model are emphasized with bold orange text. These results are presented for datasets on which the algorithm converged (using $\delta \leq 1 \times 10^{-3}$) prior to reaching the maximum number of iterations (200) for all three models.

adjusting the initial parameter values resolved this issue. Point estimates were ultimately calculate for all 1000 simulated datasets in each setting. We also encountered a few issues with standard error estimation. In all three settings, we occasionally estimated invalid (negative) variances for the measurement submodel parameter $\sigma^2_{\epsilon_4}$. This issue occurred for one non-ILD dataset in setting 1, 10 in setting 2, and 18 in setting 3, out of a total of 1,000 non-ILD datasets in each setting. Furthermore, for three datasets in setting 3, the estimated Hessian (approximated using the `optimHess` function from the stats package; R Core Team, 2022) was not invertible and so we were unable to get standard error estimates for any parameters. In the results presented in this section, we ignore the invalid and missing variance estimates. It is likely that the infrequent longitudinal measurements lead to issues with the numerical approximation used to calculate the Hessian. These challenges are not entirely unexpected, as our method was developed for the ILD setting and so we did not anticipate it to work perfectly in the non-ILD setting. Estimation in the non-ILD setting is challenging, as the data contain very little information on correlation, which is needed to estimate the parameters (particularly θ) well.

Supplementary Figure 1 shows the relative bias of parameter estimates across 1,000 simulated datasets under each true parameter setting. We see that the relative bias is low for most parameters, as in the ILD setting, but bias is higher for the OU process parameter θ , compared to the ILD setting. As mentioned in the main text (Section 4.2), the OU process parameter θ captures the correlated change in the longitudinal latent process. Since our model assumes that the latent process can be reliably measured through the observed longitudinal outcomes, measurements of the longitudinal outcomes must be close enough together that the correlation of the OU process is captured. We see that in the non-ILD setting, the frequency of measurements is low enough to lead to bias. The correlation half-life of the OU process can provide some insight into what values of θ one may reasonably expect to recover with low bias given the gaps between measurement occasions.

Supplementary Figure 2 summarizes a comparison of the standard deviation of the point estimates (across the 1,000 simulated datasets) and the average estimated standard error. We see that the average standard error is close to the empirical standard deviation for most parameters. For some elements of θ, the average standard error deviates from the empirical

**Param.**	**Truth**	**Relative bias (x 100)**	**RMSE**	**Empirical SD**	**SE**	**Coverage rate (%)**
λ_1	1.16	-0.31	0.05	0.054	0.054	94.2
λ_2	1.74	-0.36	0.07	0.072	0.070	94.0
λ_3	-0.39	-0.19	0.03	0.033	0.033	95.0
λ_4	1.97	-0.06	0.09	0.090	0.092	94.9
$\sigma^2_{u_1}$	1.10	-0.73	0.14	0.145	0.148	95.0
$\sigma^2_{u_2}$	1.30	-0.41	0.21	0.209	0.208	94.3
$\sigma^2_{u_3}$	1.40	-0.71	0.15	0.151	0.152	93.4
$\sigma^2_{u_4}$	0.90	-1.80	0.20	0.199	0.199	94.6
$\sigma^2_{e_1}$	0.60	-0.76	0.05	0.046	0.045	94.7
$\sigma^2_{e_2}$	0.50	-0.66	0.07	0.074	0.074	94.4
$\sigma^2_{e_3}$	0.40	-0.12	0.02	0.024	0.024	94.4
$\sigma^2_{e_4}$	0.70	-3.44	0.24	0.242	0.246	94.7
$\theta_{OU_{11}}$	1.00	2.51	0.49	0.487	0.527	96.5
$\theta_{OU_{21}}$	3.91	3.07	1.14	1.132	1.562	92.7
$\theta_{OU_{12}}$	0.61	5.43	0.57	0.569	0.608	95.2
$\theta_{OU_{22}}$	5.00	3.68	1.41	1.403	1.969	93.0
$\sigma_{OU_{11}}$	1.04	-0.66	0.09	-	-	-
$\sigma_{OU_{22}}$	2.03	1.01	0.32	-	-	-

Supplementary Table 8: Non-ILD setting 1. Across the 1000 simulation replicates, we report the average relative bias ((estimate - truth) / truth), root mean squared error (RMSE), empirical standard deviation (SD) of the point estimates, the average estimated standard error (SE), and the coverage rate of the 95% confidence intervals. Note that average relative bias is scaled up by a factor of 100 and that coverage rate is reported as a percent.

standard deviation, reflecting the additional challenges of the non-ILD setting.

In Supplementary Tables 8, 9, and 10, we summarize average relative bias (reported as (estimate - truth) / truth $\times$ 100), RMSE, the empirical standard deviation of the point estimates across simulation replicates, the average standard error, and the coverage rates of 95% confidence intervals.

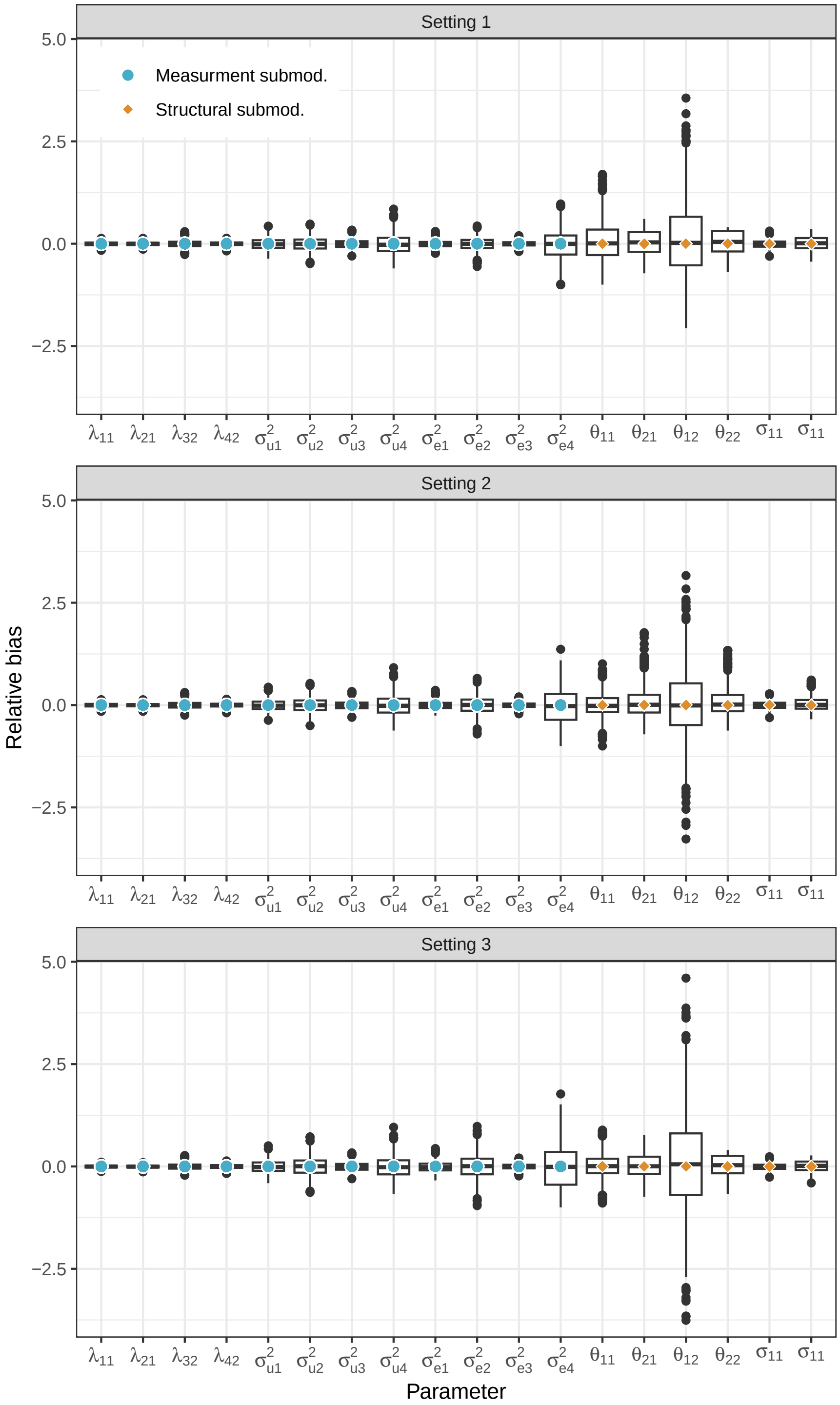

Supplementary Figure 1: Results from non-ILD simulation study. Relative bias of parameter estimates from the block coordinate descent algorithm for the three different settings in which the true OU process differs. Relative bias is calculated as (estimate - truth) / truth and is summarized across the 1000 simulated datasets with box plots. The colored dots indicate 0 bias.

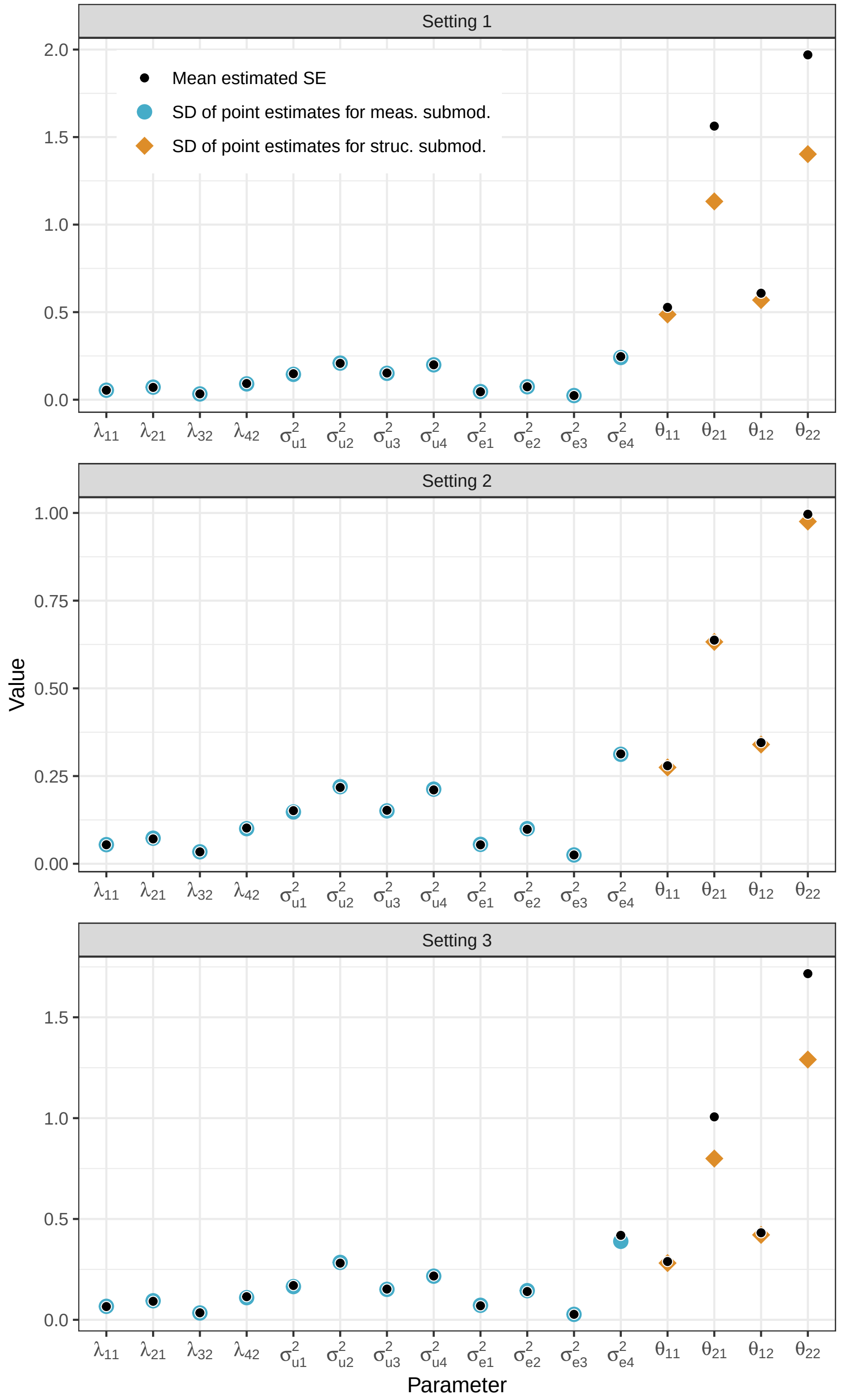

Supplementary Figure 2: Results from non-ILD simulation study. Comparison of estimated standard errors (from Fisher information) and standard deviation of point estimates. The similarity of the standard error estimates and empirical standard deviation suggests that the standard errors are of appropriate size for all parameters except some elements of θ.

**Param.**	**Truth**	**Relative bias (x 100)**	**RMSE**	**Empirical SD**	**SE**	**Coverage rate (%)**
λ_1	1.20	-0.30	0.05	0.054	0.054	94.1
λ_2	1.80	-0.41	0.07	0.073	0.071	93.9
λ_3	-0.40	-0.20	0.03	0.034	0.034	94.1
λ_4	2.00	-0.04	0.10	0.100	0.102	93.9
$\sigma^2_{u_1}$	1.10	-0.77	0.15	0.148	0.151	95.2
$\sigma^2_{u_2}$	1.30	-0.37	0.22	0.220	0.218	94.1
$\sigma^2_{u_3}$	1.40	-0.71	0.15	0.151	0.152	93.7
$\sigma^2_{u_4}$	0.90	-1.45	0.21	0.211	0.210	94.6
$\sigma^2_{e_1}$	0.60	-0.92	0.06	0.055	0.054	94.3
$\sigma^2_{e_2}$	0.50	-0.39	0.10	0.100	0.098	94.1
$\sigma^2_{e_3}$	0.40	-0.15	0.03	0.025	0.025	93.8
$\sigma^2_{e_4}$	0.70	-4.81	0.31	0.313	0.313	93.4
$\theta_{OU_{11}}$	1.00	0.42	0.28	0.275	0.279	96.2
$\theta_{OU_{21}}$	1.80	6.72	0.65	0.634	0.638	93.9
$\theta_{OU_{12}}$	0.40	0.70	0.34	0.341	0.345	94.8
$\theta_{OU_{22}}$	3.00	7.40	1.00	0.980	0.997	93.5
$\sigma_{OU_{11}}$	1.25	-0.36	0.11	-	-	-
$\sigma_{OU_{22}}$	2.00	2.48	0.32	-	-	-

Supplementary Table 9: Non-ILD setting 2. Across the 1000 simulation replicates, we report the average relative bias ((estimate - truth) / truth), root mean squared error (RMSE), empirical standard deviation (SD) of the point estimates, the average estimated standard error (SE), and the coverage rate of the 95% confidence intervals. Note that average relative bias is scaled up by a factor of 100 and that coverage rate is reported as a percent.

Param.	Truth	Relative bias (x 100)	RMSE	Empirical SD	SE	Coverage rate (%)
λ_1	1.88	-0.35	0.07	0.067	0.066	94.4
λ_2	2.83	-0.44	0.10	0.094	0.092	93.6
λ_3	-0.45	-0.06	0.03	0.034	0.035	94.7
λ_4	2.25	-0.11	0.11	0.110	0.115	92.5
$\sigma^2_{u_1}$	1.10	-0.73	0.16	0.165	0.170	95.9
$\sigma^2_{u_2}$	1.30	-0.05	0.28	0.285	0.281	94.6
$\sigma^2_{u_3}$	1.40	-0.71	0.15	0.151	0.152	93.8
$\sigma^2_{u_4}$	0.90	-1.74	0.22	0.216	0.217	94.5
$\sigma^2_{e_1}$	0.60	-1.08	0.07	0.072	0.070	93.7
$\sigma^2_{e_2}$	0.50	0.10	0.14	0.145	0.140	93.3
$\sigma^2_{e_3}$	0.40	-0.25	0.03	0.027	0.027	93.7
$\sigma^2_{e_4}$	0.70	-4.19	0.39	0.390	0.417	90.8
$\theta_{OU_{11}}$	1.00	0.88	0.28	0.281	0.289	95.4
$\theta_{OU_{21}}$	2.78	3.01	0.80	0.800	1.006	94.0
$\theta_{OU_{12}}$	0.36	4.41	0.42	0.421	0.432	94.5
$\theta_{OU_{22}}$	5.00	3.54	1.30	1.290	1.716	93.0
$\sigma_{OU_{11}}$	1.27	-0.36	0.09	-	-	-
$\sigma_{OU_{22}}$	2.66	0.86	0.36	-	-	-

Supplementary Table 10: Non-ILD setting 3. Across the 1000 simulation replicates, we report the average relative bias ((estimate - truth) / truth), root mean squared error (RMSE), empirical standard deviation (SD) of the point estimates, the average estimated standard error (SE), and the coverage rate of the 95% confidence intervals. Note that average relative bias is scaled up by a factor of 100 and that coverage rate is reported as a percent.

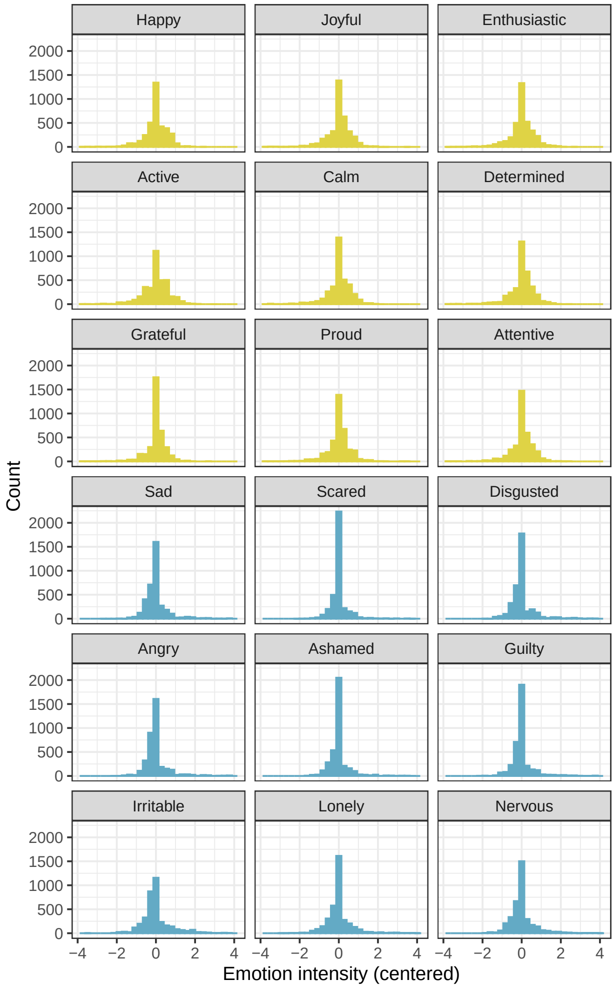

Supplementary Figure 3: Marginal distribution of responses to emotion items across all individuals in the mHealth study. Positive emotions are in yellow and negative emotions are in blue.

Section B

B.1 Application to mHealth emotion data

We provide the marginal distribution of the reponses to the emotion items in Supplementary Figure 3.

B.1.1 OUF model with one factor

In this model, we assume that a single latent factor generates all observed emotions of happy, joyful, enthusiastic, active, calm, determined, grateful, proud, attentive, sad, scared, disgusted, angry, ashamed, guilty, irritable, lonely, and nervous. We plot the point estimates from this model in Supplementary Figure 4. Using these estimated parameters, we calculate the auto-correlation half-life of this latent factor as approximately 27 days. This model has

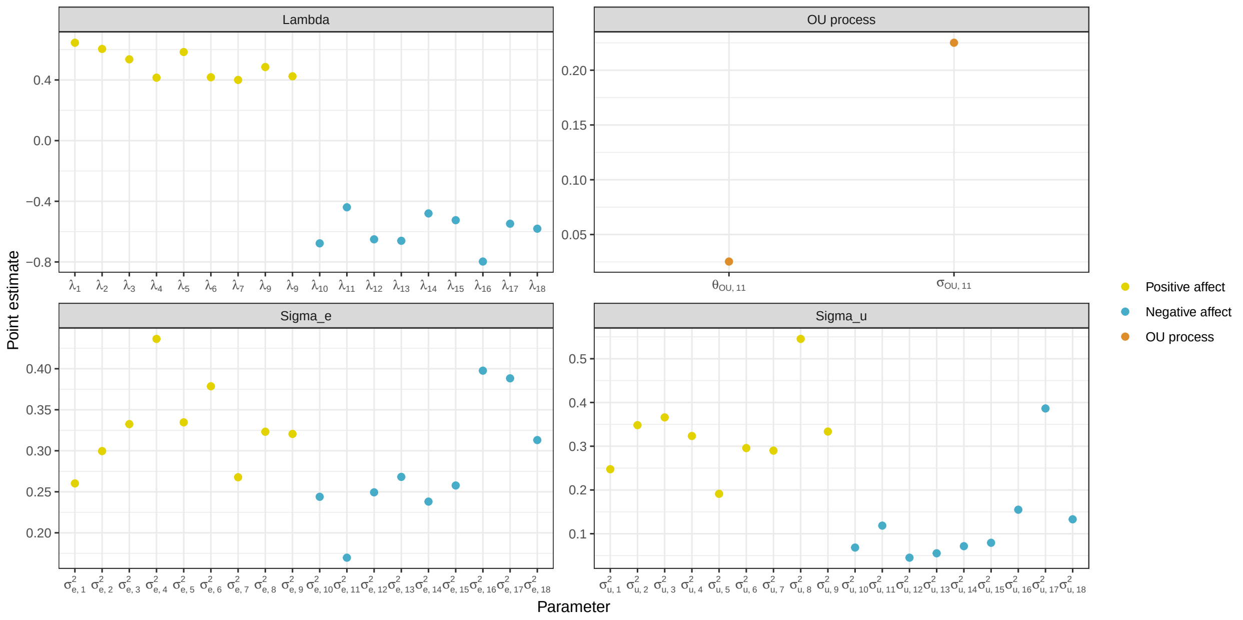

Supplementary Figure 4: Point estimates for each of the parameter matrices in our one-factor OUF model. Because we assume structural zeros in the loadings matrix are known, each emotion has only a single loading. Parameter subscripts 1-18 correspond to the emotions as follows: 1 = happy, 2 = joyful, 3 = enthusiastic, 4 = active, 5 = calm, 6 = determined, 7 = grateful, 8 = proud, 9 = attentive, 10 = sad, 11 = scared, 12 = disgusted, 13 = angry, 14 = ashamed, 15 = guilty, 16 = irritable, 17 = lonely, 18 = nervous.

a total of 56 parameters, along with one constraint, which we use when calculating AIC and BIC.

B.1.2 OUF model with two factors

In this model, we assume that two latent factors generate the observed emotions. The latent factors represent positive affect (which underlies happy, joyful, enthusiastic, active, calm, determined, grateful, proud, and attentive) and negative affect (which underlies sad, scared, disgusted, angry, ashamed, guilty, irritable, lonely, and nervous). Results from this fitted model are available in Section 5 of the main text. This model has a total of 60 parameters, along with two constraints, which we use when calculating AIC and BIC.

B.1.3 OUF model with three factors

We assume that three latent emotional states underlie the emotions observed during this study. The emotions load on to the latent factors as follows:

1. enthusiastic, proud, active, calm, determined, attentive, grateful [η_1 = high arousal positive affect]

2. calm, happy, joyful [η_2 = no-to-low arousal positive affect]

Positive affect items	Arousal	Citation
calm	no-to-low	McManus (2019), Gilbert (2008), Remington (2000)
grateful	high	Reisenzein (1994)
proud	high	McManus (2019)
happy	no-to-low	Remington (2000)
joyful	no-to-low	Remington (2000)
enthusiastic	high	McManus (2019), Gilbert (2008), Remington (2000)
active	high	McManus (2019), Gilbert (2008), Remington (2000)
determined	high	McManus (2019)
attentive	high	McManus (2019)

Supplementary Table 11: Behavioral science literature supporting the division of the positive emotions into two groups representing no-to-low arousal positive affect and high arousal positive affect.

3. sad, scared, disgusted, angry, ashamed, guilty, irritable, lonely, nervous [η_3 = negative affect]

We use behavioral science literature and theory—namely the circumplex model of emotion—to inform the division of the positive affect emotions into groups representing high arousal positive affect and no-to-low arousal positive affect (see Gilbert et al. (2008), McManus et al. (2019), Reisenzein (1994), and Remington et al. (2000)). Literature supporting the placement of each positive affect emotion is summarized in Supplementary Table 11. Happy and joyful are also commonly placed midway between high and low arousal in the circumplex model of emotion (see Remington et al. (2000)) and so we chose to assess the fit of the OUF model when these emotion items load onto the latent factor representing no-to-low arousal positive affect. This model converged after 211 block iterations and we present point estimates in Supplementary Figure 5. This model has a total of 66 parameters, along with three constraints, which we use when calculating AIC and BIC.

Section C

C.1 Estimation algorithm

C.1.1 Parameter initialization

Due to the complexity of our model, our estimation algorithm is sensitive to the choice of initial estimates. Here we present an approach to estimating reasonable starting values based on simple existing models prior to maximizing the entire likelihood.

1. To initialize the **measurement submodel parameters**, fit a standard cross-sectional factor model to the data collapsed across time (do not include a random intercept but do assume that the positions of the non-zero loadings are known).

2. Using this fitted factor model, estimate the factor scores (predicted values for η_1 and η_2).

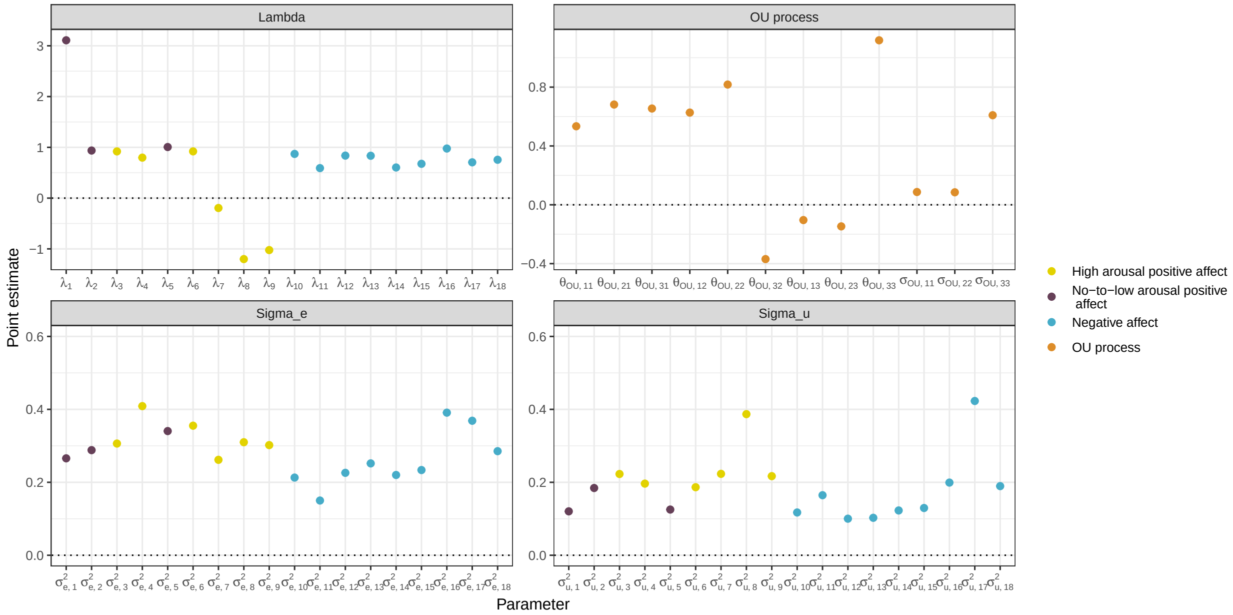

Supplementary Figure 5: Point estimates for each of the parameter matrices in our three-factor OUF model. Because we assume structural zeros in the loadings matrix are known, each emotion has only a single loading. Parameter subscripts 1-18 correspond to the emotions as follows: 1 = happy, 2 = joyful, 3 = enthusiastic, 4 = active, 5 = calm, 6 = determined, 7 = grateful, 8 = proud, 9 = attentive, 10 = sad, 11 = scared, 12 = disgusted, 13 = angry, 14 = ashamed, 15 = guilty, 16 = irritable, 17 = lonely, 18 = nervous.

3. Fit four separate linear mixed effects models—one for each of the observed outcomes, $Y_1, ..., Y_K$—including the factor scores as fixed effects and a random intercept for subject. We do not include a fixed effect intercept in these models. For outcome $k = 1, ..., K$, subject $i = 1, ..., N$, and measurement occasion $j = 1, ..., n_i$, the mixed model is given by
$$Y_{kij} = \lambda_k \eta_i(t_j) + u_{k0i} + \epsilon_{kij}$$
where $u_{k0i} \sim N(0, \sigma^2_{u_k})$ and $\epsilon_{kij} \sim N(0, \sigma^2_{\epsilon_k})$.

4. From each of these K mixed models, extract estimates of the coefficient for the fixed effect, the variance for the random intercept, and the residual variance. Use the coefficients of the fixed effects to initialize the non-zero elements of Λ and the variance estimates to initialize the diagonal components of Σ_u and Σ_ϵ. In some cases, the estimated variances were very small, so a lower limit of 0.1 was set for the initial parameter values to avoid extremely negative estimates after logging. We also set the same lower bound for initial values of the elements in the loadings matrix.

5. To initialize the **structural submodel parameters**, we add a term for white noise to the OU process likelihood. This noise term will absorb some of the extra variability in the predicted factor scores and allow for more stable estimation. Let Γ_i be white noise, then $\eta_i \sim N(0, \Psi_i + \Gamma_i)$ where Γ_i is a diagonal matrix (of the same dimension as OU covariance matrix Ψ_i) with constant but unknown diagonal γ. We then maximize this likelihood and use the estimated OU process parameter values as initial values, restricting the maximum initial values of the diagonals of θ_{OU} to be less than 7. This maximum helps deal with instability in the initial estimate of θ.

C.1.2 Maximization of the marginal log-likelihood

To maximize the log-likelihood, we use quasi-Newton optimizers as implemented in the `stats` package in `R` (R Core Team, 2022). To prevent the parameter estimates from diverging to infinite values, we set the maximum allowed step size to 10.

Using the initial parameter values estimated via the approach described in the previous section, we iteratively update measurement and structural submodel parameter estimates in blocks:

1. Initialize estimates: $\Lambda^{(0)}, \Sigma_u^{(0)}, \Sigma_\epsilon^{(0)}, \theta^{(0)}, \sigma^{(0)}$. Measurement submodel parameters are always initialized empirically; for structural submodel parameters, two sets of initial estimates are considered—an empirical set of values estimated as described above and a default set of values that are based on a reasonable guess. The set of values that corresponds to the higher log-likelihood is used.

2. Set $r = 1$ and $\delta = 0$. While $r \leq 200$ and $\delta = 0$,

   (a) Update block of **measurement submodel parameters**:
$$\Lambda^{(r)}, \Sigma_u^{(r)}, \Sigma_\epsilon^{(r)} = \underset{\Lambda, \Sigma_u, \Sigma_\epsilon}{argmax} \{ logL(Y | \theta^{(r-1)}, \sigma^{(r-1)}) \}.$$

We solve this iteratively using `nlm` (R Core Team, 2022) and analytic gradients with convergence criteria set to `gradtol` $= max(1 \times 10^{-4}/10^{r}, 1 \times 10^{-8})$ and `steptol` $= max(1 \times 10^{-4}/10^{r}, 1 \times 10^{-8})$. `gradtol` is the tolerance for the scaled gradient and `steptol` is the tolerance for parameter estimates across iterations. We model the first element of the loadings matrix and the variance parameters on the log scale, since all of these estimates are required to be positive.

(b) Update block of **structural submodel parameters**:

$$\theta^{(r)}, \sigma^{(r)} = \underset{\theta,\sigma}{argmax}\left\{logL(Y|\Lambda^{(r)}, \Sigma_u^{(r)}, \Sigma_\epsilon^{(r)})\right\}.$$

We solve this iteratively using `nlminb` and numeric approximations to the gradients. For estimates of θ, the diagonal elements must be positive and the matrix must have eigenvalues with positive real parts. The eigenvalue constraint is implemented by adding a negative penalty term to the likelihood for proposed values of θ that do not satisfy this constraint. The diagonal element of σ are estimated on the log scale, since they are required to be positive.

(c) Check for block-wise convergence: Let Θ be a vector containing all elements of Λ, Σ_u, Σ_ϵ, θ, and σ. Then, calculate

$$\delta = \max\left\{I\left\{|\Theta^{(r)} - \Theta^{(r-1)}|/\Theta^{(r)} < 10^{-6}\right\}, I\left\{logL(\Theta^{(r)}|Y) - logL(\Theta^{(r-1)}|Y) < 10^{-6}\right\}\right\}$$

where all operations on Θ are element-wise.

(d) Rescale OU process parameters so stationary variance is equal to 1 using Equation 5 in the main paper.

(e) Update r: $r = r + 1$

3. Estimate standard errors using a numerical approximation to the Hessians of the joint negative log-likelihood for $\Lambda^{(r)}, \Sigma_u^{(r)}, \Sigma_\epsilon^{(r)}, \theta^{(r)}$ at the current parameter values. Rather than rescaling the OU parameters so the stationary variance is equal to 1, we assume that σ is a function of θ. See Section A.6 of the supplementary material for further description of this function. The numeric approximation to the Hessians is carried out using the `optimHess` function in the `stats` package.

4. Estimate confidence interval for OU process parameter σ based on a parametric bootstrap of θ.

C.2 Comparison with Tran et al. (2021)

To illustrate the computational benefits of our proposed block coordinate descent algorithm for estimation relative to the Bayesian approach taken in Tran et al. (2021), we apply both methods to simulated ILD. Because we only consider continuous outcomes in this work, we slightly modify the original model proposed in Tran et al. (2021) and do not estimate the additional parameters used to account for non-continuous outcomes. Tran et al. (2021) also

consider two different sets of constraints on the OU process drift matrix (denoted here as θ_{OU}); we use the set of constraints that specify the eigenvalues of θ_{OU} to have positive real parts.

We use the same ILD simulation set-up as described in the main text (Section 4.1) with the true OU process parameters corresponding to setting 1 (Section A.7). We make one modification to the true values of the loadings parameters: we restrict *all* elements of the loadings matrix to be positive. This restriction means that $\lambda_3 = 0.4$, rather than the original $\lambda_3 = -0.4$. We make this assumption in order to make identification of parameters more straightforward in this comparison of methods.

We generate 100 replicates of the simulated dataset and fit the OUF model using our proposed estimation algorithm and the algorithm proposed in Tran et al. (2021). Tran et al. (2021) use a slightly different parameterization of the OU process than we use in this work. In our implementation of the OU process, we restrict the volatility parameter matrix, σ_{OU}, to be a diagonal matrix. Although Tran et al. (2021) do not make this assumption, there is still a one-to-one correspondence between the set of parameters estimated in our work and the set of posterior estimates resulting from their Bayesian method. As a result of these differences in parameters, we do not report estimates of σ_{OU} in the plot below and instead present parameter estimates for ρ, which is the stationary correlation between η_1 and η_2. Tran et al. estimate this parameter directly and we can calculate an estimate for it using $\hat{\theta}_{OU}$ and $\hat{\sigma}_{OU}$.

When applying the Bayesian approach, we use our proposed empirical approach to initializing parameter values, assume 4 chains, and allow the sampler to run for 2,000 iterations. We discard the first half of these samples as burn-in. The computation time of both approaches—excluding time required to compute initial parameter estimates—is shown in Supplementary Figure 6. Computing resources are the same across all replicates; we use 4 cores with a total of 4GB of memory for each replicate (this allows gradients to be evaluated or chains to be sampled in parallel, depending on the method). We find that our approach, on average, takes approximately 5% of the time required by the method in Tran et al. (2021).

Point estimates for both estimation approaches are shown in Supplementary Figure 7. We present the posterior means for each parameter across the 100 simulated datasets as estimated using the method from Tran et al. (2021); maximum likelihood estimates resulting from our block coordinate descent algorithm are also summarized across the 100 simulated datasets. We also report the average relative bias (where relative bias is calculated as (estimate - truth) / truth), RMSE, and the coverage probability for comparison across the two methods in Supplementary Table 12. We find that overall, performance is similar for most parameters, but the relative bias and RMSE is substantially lower for the OU process parameter θ estimated using our method compared to the Bayesian alternative. It is important to note, however, that the posterior point estimates from the Bayesian approach may be improved by running the sampling algorithm for additional iterations; we limit the MCMC algorithm to 2,000 iterations as our goal is to emphasize the difference in computation time.

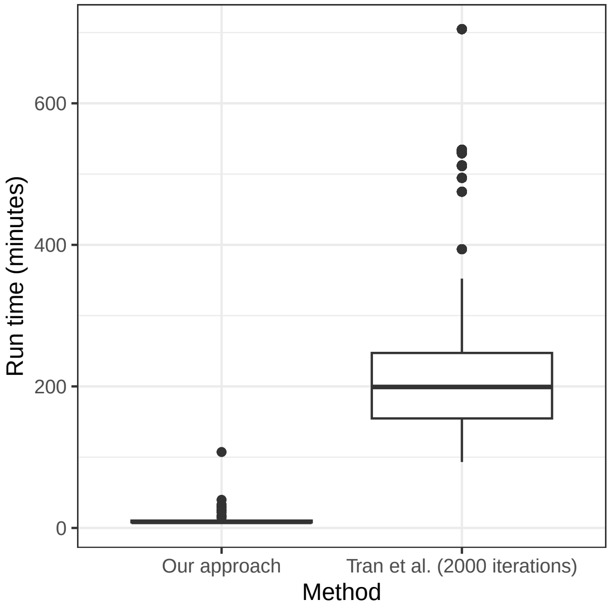

Supplementary Figure 6: Computation time (in minutes) for our estimation algorithm and the Bayesian estimation method proposed in Tran et al. (2021). Box plots summarizes the computation time required to fit the OU factor model using both approaches across 100 simulated datasets. Time required to compute initial parameter estimates is not included in the total above. For our approach, the total time includes both the time required to carry out the block coordinate descent algorithm plus the time required to estimate standard errors.

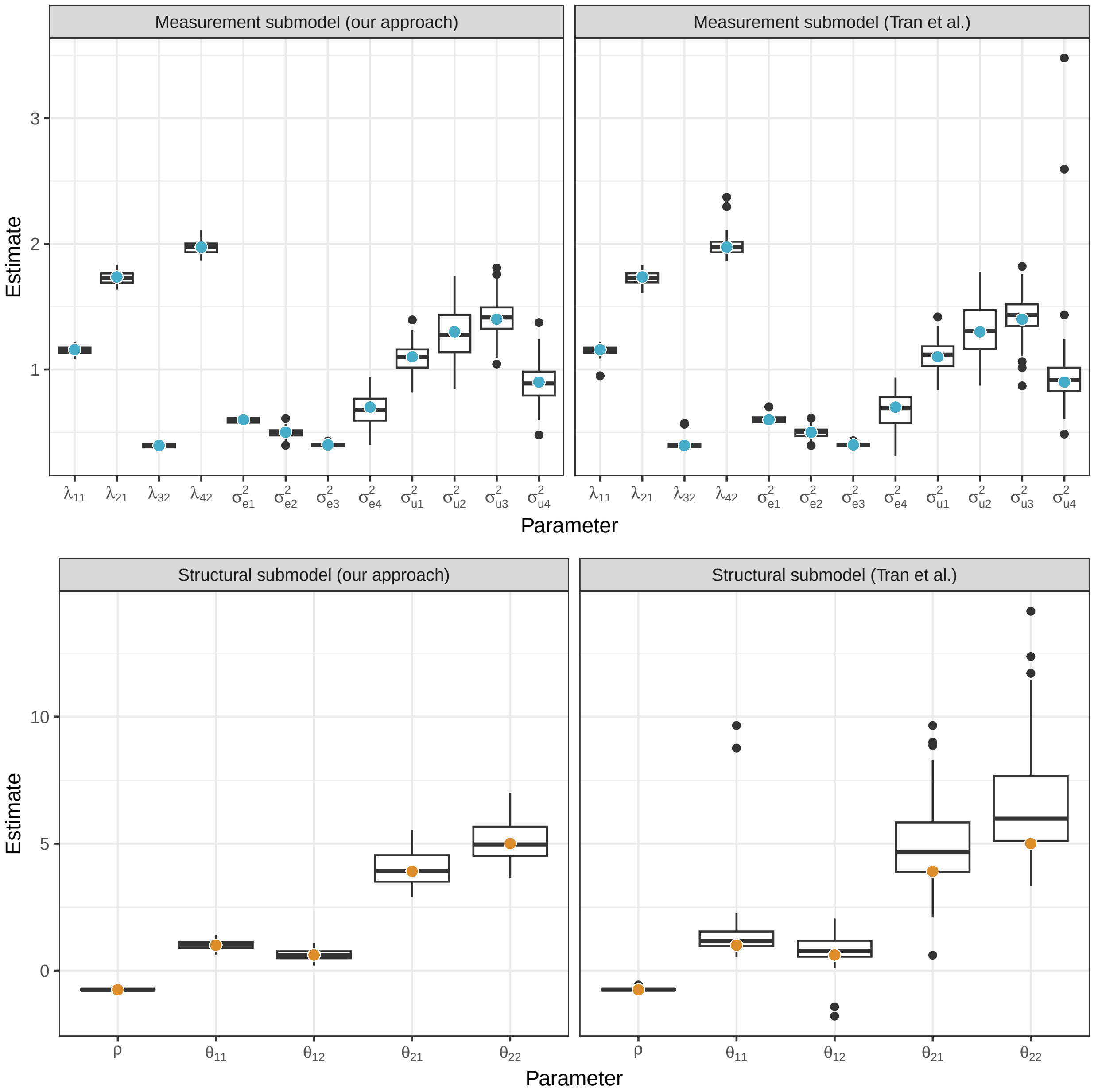

Supplementary Figure 7: Final parameter estimates from the block coordinate descent algorithm and the Bayesian estimation method proposed in Tran et al. (2021). For the Bayesian method, posterior means are used for point estimates. Each box plot summarizes point estimates across the 100 simulated datasets. True parameter values are indicated with colored dots.

	Avg. relative bias x 100		RMSE		Coverage prob. (%)	
Parameter	Our approach	Tran et al.	Our approach	Tran et al.	Our approach	Tran et al.
λ_1	-0.57	-0.66	0.03	0.04	97	96
λ_2	-0.39	-0.41	0.04	0.05	94	96
λ_3	-0.46	0.50	0.02	0.03	94	92
λ_4	-0.15	0.46	0.05	0.08	92	94
$\sigma^2_{u_1}$	-1.46	0.83	0.11	0.12	95	96
$\sigma^2_{u_2}$	-1.71	1.37	0.20	0.20	91	92
$\sigma^2_{u_3}$	0.75	1.71	0.15	0.16	93	94
$\sigma^2_{u_4}$	-0.36	7.10	0.15	0.35	93	92
$\sigma^2_{e_1}$	-0.43	0.28	0.02	0.02	95	97
$\sigma^2_{e_2}$	-0.73	-0.04	0.04	0.04	94	96
$\sigma^2_{e_3}$	-0.04	0.49	0.01	0.01	97	94
$\sigma^2_{e_4}$	-1.96	-2.89	0.12	0.14	96	96
$\theta_{OU_{11}}$	1.02	40.47	0.17	1.25	96	95
$\theta_{OU_{21}}$	2.49	28.02	0.64	1.92	93	88
$\theta_{OU_{12}}$	1.89	37.94	0.19	0.62	96	96
$\theta_{OU_{22}}$	2.68	32.27	0.81	2.64	94	86
ρ	-0.02	-0.81	0.02	0.03		97

Supplementary Table 12: Comparison of estimates from fitting the dynamic factor model using our proposed block-coordinate descent algorithm and the Bayesian approach presented in Tran et al. (2021). We report average relative bias ((estimate - truth) / truth), root mean squared error (RMSE), and coverage probabilities for models fit using each method to the same 100 simulated datasets. Note that relative bias is scaled up by a factor of 100 and coverage probability is reported as a percent.

Model	AIC	BIC	Free parameters
Random slope/random intercept model	133,531	134,478	108
Dynamic factor model with 1 factor	123,309	123,791	55
Dynamic factor model with 2 factors	**121,069**	**121,577**	58
Dynamic factor model with 3 factors	124,957	125,509	63

Supplementary Table 13: AIC, BIC, and numbers of free parameters for mixed models and dynamic factor models fit to the motivating mHealth data. The lowest (best) value of AIC and BIC is indicated with bold text. For the dynamic factor models, the number of free parameters takes into account the identifiability constraints.

C.3 Comparison with standard mixed models

To provide an additional comparison of the dynamic factor model with existing methods, we compare our model's fit to a fitted model with both random slopes and random intercepts. We fit a mixed model with a correlated random slope and random intercept separately to each of the 18 outcomes. Assuming the outcomes are independent, we can multiply the likelihoods of all 18 models and use this quantity to calculate the models' combined AIC and BIC, which we then compare to those from our dynamic factor models from the original paper; see Supplementary Table 13. In Supplementary Table 13, we also report the number of free parameters in each model. For the dynamic factor models, the free parameters correspond to the total number of parameters minus the constraints. AIC and BIC both indicate that all three dynamic factor models fit better than the random slope/random intercept model, which has nearly twice as many parameters as the best fitting factor model. Among the factor models considered, AIC and BIC indicate that the two-factor model fits the best.

This result emphasizes the importance of capturing the correlation between related outcomes, which the dynamic factor model does by summarizing the outcomes as a smaller number of correlated latent factors. In this comparison, the importance of modeling the correlation outweighs any information lost due to the dimension-reduction aspect of the dynamic factor model.

References

Gilbert, P., McEwan, K., Mitra, R., Franks, L., Richter, A., & Rockliff, H. (2008). Feeling safe and content: A specific affect regulation system? relationship to depression, anxiety, stress, and self-criticism. *The Journal of Positive Psychology*, *3*(3), 182–191.

McManus, M., Siegel, J., & Nakamura, J. (2019). The predictive power of low-arousal positive affect. *Motivation and Emotion*, *43*, 130–144.

R Core Team. (2022). *R: A language and environment for statistical computing*. R Foundation for Statistical Computing. Vienna, Austria. https://www.R-project.org/

Reisenzein, R. (1994). Pleasure-arousal theory and the intensity of emotions. *Journal of Personality and Social Psychology*, *67*(3), 525–539.

Remington, N. A., Fabrigar, L. R., & Visser, P. S. (2000). Reexamining the circumplex model of affect. *Journal of Personality and Social Psychology*, *79*(2), 286–300.

Tran, T. D., Lesaffre, E., Verbeke, G., & Duyck, J. (2021). Latent Ornstein-Uhlenbeck models for Bayesian analysis of multivariate longitudinal categorical responses. *Biometrics*, *77*(2), 689–701. https://doi.org/10.1111/biom.13292

Vatiwutipong, P., & Phewchean, N. (2019). Alternative way to derive the distribution of the multivariate Ornstein–Uhlenbeck process. *Advances in Difference Equations*, *276*.
